# Identification and Correction of Mechanisms Underlying Inherited Blindness in Human iPSC-Derived Optic Cups

**DOI:** 10.1016/j.stem.2016.03.021

**Published:** 2016-06-02

**Authors:** David A. Parfitt, Amelia Lane, Conor M. Ramsden, Amanda-Jayne F. Carr, Peter M. Munro, Katarina Jovanovic, Nele Schwarz, Naheed Kanuga, Manickam N. Muthiah, Sarah Hull, Jean-Marc Gallo, Lyndon da Cruz, Anthony T. Moore, Alison J. Hardcastle, Peter J. Coffey, Michael E. Cheetham

**Affiliations:** 1 Ocular Biology and Therapeutics, UCL Institute of Ophthalmology, 11-43 Bath Street, London EC1V 9EL, UK; 2 Moorfields Eye Hospital, 162 City Road, London EC1V 2PD, UK; 3 Maurice Wohl Clinical Neurosciences Institute, Institute of Psychiatry, Psychology, and Neuroscience, Kings College London, London SE5 9NU, UK

## Abstract

Leber congenital amaurosis (LCA) is an inherited retinal dystrophy that causes childhood blindness. Photoreceptors are especially sensitive to an intronic mutation in the cilia-related gene CEP290, which causes missplicing and premature termination, but the basis of this sensitivity is unclear. Here, we generated differentiated photoreceptors in three-dimensional optic cups and retinal pigment epithelium (RPE) from iPSCs with this common CEP290 mutation to investigate disease mechanisms and evaluate candidate therapies. iPSCs differentiated normally into RPE and optic cups, despite abnormal CEP290 splicing and cilia defects. The highest levels of aberrant splicing and cilia defects were observed in optic cups, explaining the retinal-specific manifestation of this CEP290 mutation. Treating optic cups with an antisense morpholino effectively blocked aberrant splicing and restored expression of full-length CEP290, restoring normal cilia-based protein trafficking. These results provide a mechanistic understanding of the retina-specific phenotypes in CEP290 LCA patients and potential strategies for therapeutic intervention.

## Highlights

• Generation of 3D optic cups with opsin-expressing photoreceptors and outer segments
• A CEP290-LCA intronic mutation creates a cryptic exon that impairs ciliogenesis
• Aberrant splicing is increased in photoreceptors compared to other cell types
• Antisense oligonucleotide can block the cryptic exon and restore CEP290 function

## Graphical Abstract

**Figure:**
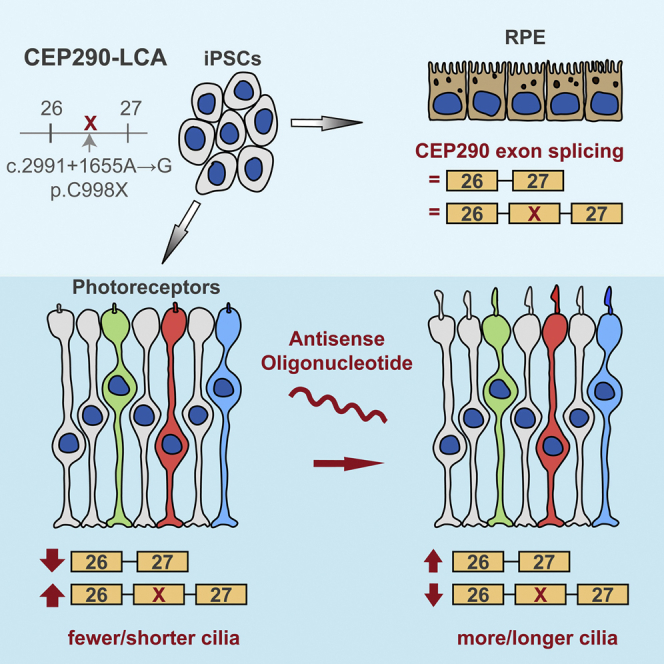

## Teaser

Parfitt et al. derived human 3D optic cup organoids to model LCA, a retinal dystrophy associated with aberrant CEP290 splicing leading to cilia defects. Retinal-specific defects result from higher aberrant CEP290 splicing in photoreceptors versus other cells, and treating cups with an antisense oligonucleotide restored CEP290 protein, function, and ciliation.

## Introduction

Primary cilia are sensory organelles that project from the cell surface in most eukaryotic cells and affect signaling cascades in response to environmental stimuli. Photoreceptors use a highly specialized sensory cilium, the “outer segment” (OS), which contains tightly stacked discs containing the photopigment opsin, to detect light. Degeneration of photoreceptors is a major cause of blindness, and Leber congenital amaurosis (LCA) is a recessively inherited form of retinal dystrophy resulting in severe visual loss in early childhood (Koenekoop, 2004). LCA affects between 1:30,000 to 1:81,000 individuals and accounts for 5% of all inherited retinal dystrophies (Koenekoop, 2004). One of the most common causative LCA genes is *CEP290* (centrosomal protein of 290 kDa; OMIM: 611755), accounting for around 15%–25% of cases (Chacon-Camacho and Zenteno, 2015, den Hollander et al., 2006, den Hollander et al., 2008).

*CEP290* (also known as *LCA10*, *BBS14*, *JBTS5*, *NPHP6*, *MKS4*, and *SLSN6*) is a 92 kb gene of 55 exons, encoding a 2479 amino acid 290 kDa protein originally described as a component of the centrosome (Andersen et al., 2003). During mitosis, CEP290 is found at the centrosome (Sayer et al., 2006), while in an interphase cell, CEP290 is located on both mother and daughter centrioles (Tsang et al., 2008). In photoreceptors, CEP290 is located in the connecting cilium (Sayer et al., 2006). During ciliogenesis, CEP290 is found at the distal end of the mother centriole that becomes part of the basal body at the base of the cilium, suggesting a role for CEP290 in cilium assembly (Chang et al., 2006, Tsang et al., 2008). Indeed, there is growing and compelling evidence for a critical function of CEP290 in ciliogenesis, as multiple studies have shown that loss of CEP290 reduces ciliogenesis (Kim et al., 2008, Sang et al., 2011, Tsang et al., 2008), without affecting cell-cycle progression. CEP290 forms a complex with CP110, another centrosomal protein, which leads to CEP290 inactivation until the cell enters quiescence, whereupon the CP110:CEP290 complex dissociates and CEP290 recruits Rab8a, a small GTPase, and triggers ciliogenesis (Tsang et al., 2008).

Mutations in *CEP290* are associated with numerous syndromic ciliopathies such as Joubert syndrome (JBTS), nephronophthisis (NPHP), Meckel-Gruber syndrome (MKS), and Senior-Loken syndrome (SLSN) (Coppieters et al., 2010). The severity of the disease and number of affected organs have been suggested to be related to the amount of residual CEP290 function (Drivas et al., 2015). A recent meta-analysis of 138 *CEP290* mutations in 250 individuals demonstrated that LCA is the most common associated phenotype (57%) and that 86% of the *CEP290* LCA patients had at least one copy of the most common *CEP290* mutation, a deep intronic c.2991+1665A>G change (Drivas et al., 2015). This mutation in intron 26 results in aberrant splicing and subsequent inclusion of a 128 base pair (bp) cryptic exon containing an immediate premature stop codon (p.C998X) between exons 26 and 27. This mutation is specifically associated with LCA retinal dystrophy, and not syndromic disease (den Hollander et al., 2006, Drivas et al., 2015). Importantly, a residual fraction of full-length *CEP290* mRNA is still spliced correctly and translated to full-length CEP290 protein. This residual CEP290 protein expression was reported to be as high as 50% of control levels in CEP290-LCA fibroblasts and has been suggested to account for the retinal-only phenotype seen in these individuals, with the remaining protein being sufficient to enable cilia function in other organs (den Hollander et al., 2006, Drivas et al., 2015). It is not clear, however, why photoreceptors would be more vulnerable to reduced CEP290 levels than other cells.

Therapeutic options for retinal dystrophies such as LCA are limited. A promising therapeutic strategy in LCA associated with the deep intronic mutation in *CEP290* is to target the aberrant splicing using antisense oligonucleotides (AONs). AONs are short, modified RNA molecules that can interfere with splicing, either to adjust levels of naturally occurring splice isoforms or to induce exon skipping of protein-truncating cryptic exons. AONs have been used in CEP290-LCA fibroblasts to provide proof-of-principle that aberrant *CEP290* splicing can be corrected, and the ciliation defect of affected cells can be restored (Collin et al., 2012, Gerard et al., 2012).

Induced pluripotent stem cells (iPSCs) are a potent technology that allows for directed differentiation toward most cell types, including retinal cell types. Using this technology with cells derived from affected patients is a powerful technique to model disease. Importantly, this provides an appropriate cellular model with the genetic mutation(s) in genomic context. We have recently used iPSC-derived retinal pigment epithelium (RPE) cells from an individual with RP2 X-linked retinitis pigmentosa to test the potential of translational readthrough-inducing drugs to bypass a stop codon and produce functional protein (Schwarz et al., 2015). Advances in iPSC-photoreceptor differentiation techniques have provided a platform for investigating the effects of patient mutations on neural retinal development and maintenance (Nakano et al., 2012, Zhong et al., 2014). In this study, we used fibroblasts from an individual with LCA caused by a homozygous c.2991+1655A>G mutation to create iPSC-derived RPE and three-dimensional (3D) optic cups. These cell types were used to probe disease mechanisms and test antisense morpholino (MO) efficacy on *CEP290* aberrant splicing. The data reveal a potential explanation for the retinal specificity associated with this mutation and proof of concept that AON therapy is effective in photoreceptors.

## Results

### LCA Fibroblasts Express Misspliced *CEP290* and Have Impaired Ciliogenesis

Fibroblasts were derived from a dermal skin biopsy of a 39-year-old male who was diagnosed with LCA as a child. The patient was homozygous for the c.2991+1665A>G change and had no signs of syndromic disease. He had no detectable ERG, and retinal imaging showed attenuated vessels, peripheral RPE atrophy, and mottling, with a centrally preserved outer nuclear layer and inner segment ellipsoid band on spectral-domain OCT scanning that corresponded to the increased hyperautofluorescent ring in the macula on autofluorescence imaging (Figure S1, available online).

**Figure 1:**
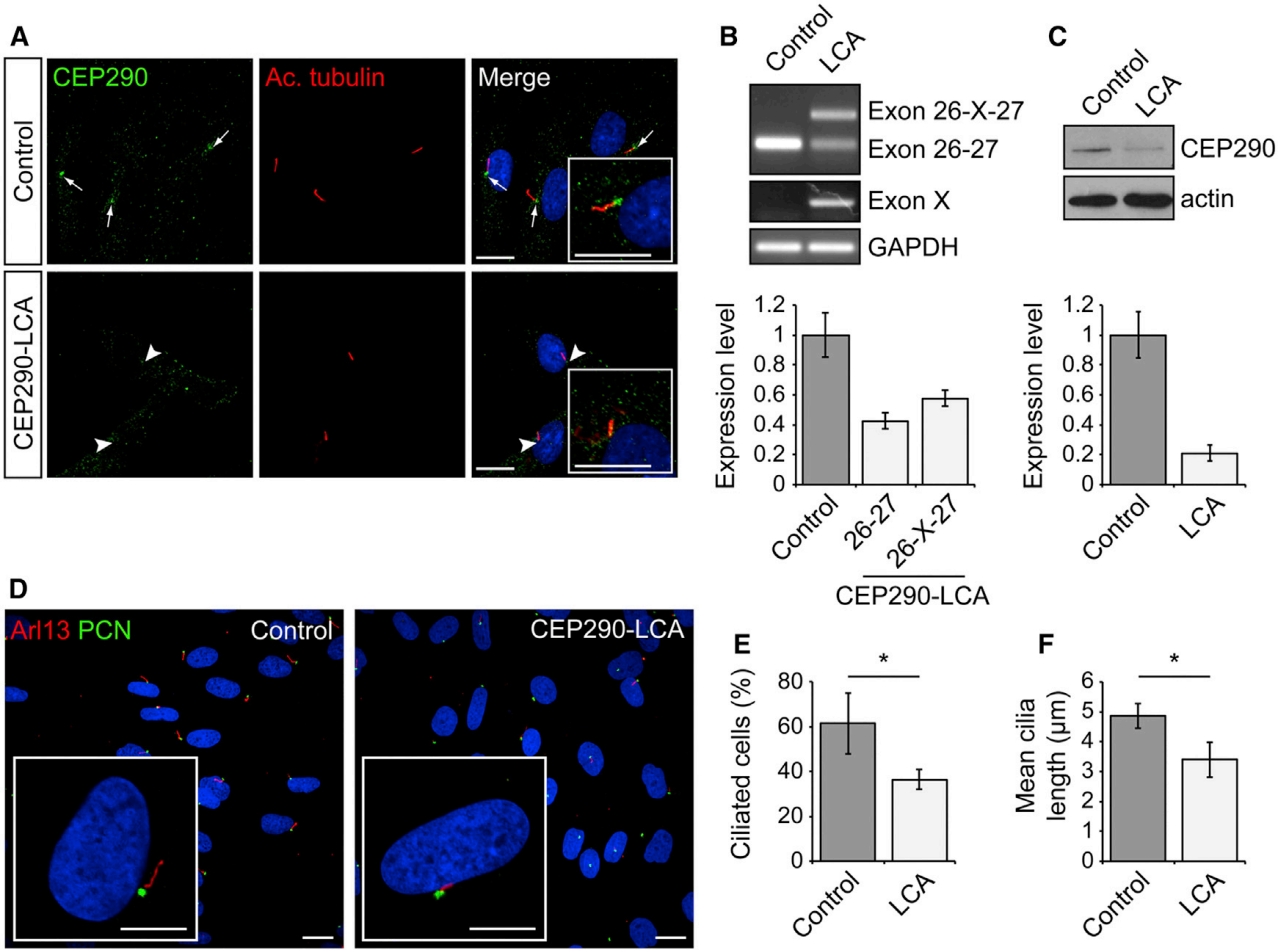
CEP290-LCA Fibroblasts Have Reduced CEP290 Levels and Ciliation (A) CEP290 (green) localization at the base of the cilia (arrow and arrowheads). Acetylated α-tubulin (Ac. tubulin; red) was used to mark the ciliary axoneme. Scale bar, 10 μm. (B) RT-PCR analysis of control and LCA cDNA using primers to exons 26 and 27, and the cryptic exon (Exon X), of *CEP290* revealed two bands in the LCA samples representing correct wild-type (WT) transcript (Exon 26-27) and a higher molecular weight band, including the cryptic exon (Exon 26-X-27), compared to the WT band alone in control. Relative quantification of band intensity is shown underneath. Values are mean ± 2 × SEM. n = 3. (C) Western blot showed a reduction in full-length wild-type CEP290 protein in LCA fibroblasts compared to controls. Quantification of band intensity relative to actin is shown underneath. (D) Representative images of cilia using cilia markers Arl13 (red), to mark the axoneme, and pericentrin (PCN; green), to mark the basal body, in control and LCA fibroblasts. Cilia appear shorter in LCA cells compared to controls (zoom panels). Scale bar, 10 μm. (E and F) Quantification of (E) ciliation of cells and (F) cilia length. Values are mean ± 2 × SEM. n = 3 biological replicates of at least 65 cells per replicate. Statistical significance was determined using Student’s t test, ^∗^p < 0.05. See also Figure S1.

In control fibroblasts, CEP290 protein was present at the base of the acetylated α-tubulin-positive cilium, as previously described (Chang et al., 2006, Valente et al., 2006). In contrast, CEP290 localization at the base of cilia was severely reduced in LCA fibroblasts (Figure 1A; arrowheads). RT-PCR analysis of LCA fibroblasts using primers spanning exons 26 and 27 of the *CEP290* gene revealed the presence of incorrectly spliced transcript containing the cryptic exon (26-X-27), in addition to the normal transcript (26-27) observed in controls (Figure 1B). Primers specific to the cryptic exon amplified a product in LCA fibroblasts only (Figure 1B). Incorrectly spliced transcript (26-X-27) represented the majority (∼60%) of total *CEP290* mRNA in LCA cells. Immunoblotting confirmed that *CEP290* missplicing leads to reduced levels of CEP290 protein compared to control fibroblasts (Figure 1C). CEP290 is known to play a critical role in the process of ciliogenesis, and cilia incidence was significantly reduced in LCA fibroblasts (Figures 1D and 1E) and the cilia present were significantly shorter (Figure 1F). Thus, we were able to detect and measure functional defects in ciliogenesis in CEP290-LCA patient cells.

### LCA-iPSC-RPE Exhibit Relatively Mild *CEP290* Aberrant Splicing and Impaired Ciliogenesis

CEP290-LCA and control fibroblasts were reprogrammed to iPSCs by electroporation with episomal vectors containing the four Yamanaka factors (Oct4, Sox2, Klf4, and Myc), Lin28, and a short hairpin RNA (shRNA) to p53 (Okita et al., 2011, Schwarz et al., 2015). Emerging iPSC colonies were isolated, expanded, and clonally selected to generate clonal lines 1 and 2 (Figures S2A–S2C). The cells formed round, tightly compacted colonies that were positive for iPSC markers (Figures S2D and S2E). The pluripotent status of the CEP290-LCA lines was confirmed by comparing gene expression at the iPSC stage and following undirected embryoid body (EB) differentiation to that of 12 embryonic and 12 iPSC reference lines using the Taqman hPSC Scorecard panel (Figures S2F and S2G) as previously described (Tsankov et al., 2015). CEP290 was present at the base of cilia in control iPSCs, but not in LCA iPSCs in which aberrant splicing of the cryptic exon 26-X-27 was detected (Figures S3A and S3B). Cilia incidence was significantly reduced in LCA iPSCs compared to control iPSCs, although the length of the remaining cilia was similar (Figures S3C–S3E).

**Figure 2:**
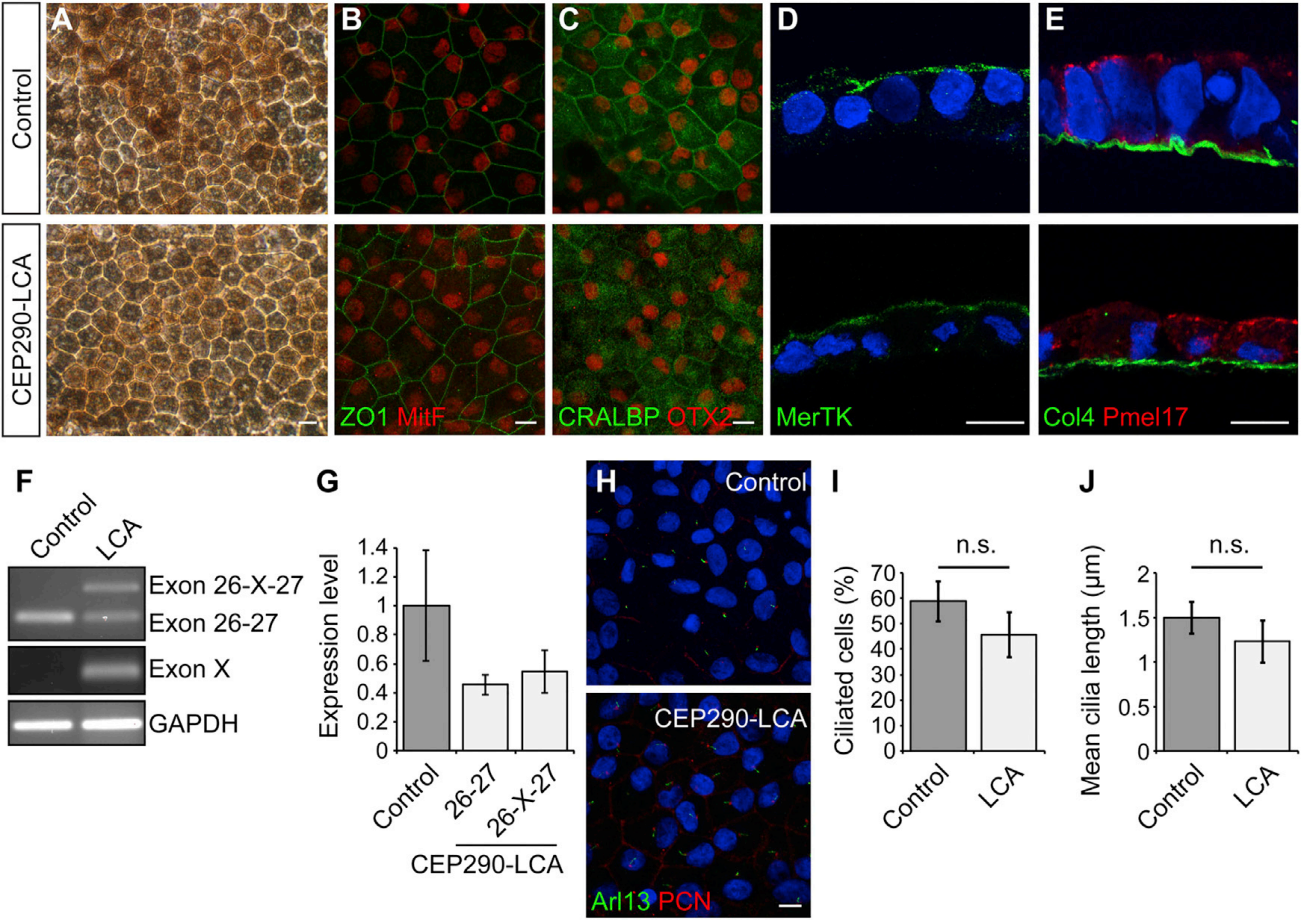
CEP290-LCA iPSC-RPE Generation (A–E) (A) Control and LCA RPE cells showed pigmentation and typical “cobblestone” appearance and expressed RPE-specific markers (B) ZO1 (green), MitF (red), (C) CRALBP (green), OTX2 (red), (D) MerTK (green), (E) Col4 (green), and Pmel17 (red). Scale bar, 20 μm. (F and G) LCA RPE cells showed expression of cryptic exon via RT-PCR analysis of *CEP290* exons 26-27 and exon X. Values are mean ± 2 × SEM. n = 3. (H) Representative immunostaining of cilia using cilia markers Arl13 (red), to mark the axoneme, and pericentrin (PCN; green), to mark the basal body, in control and LCA RPE. Scale bar, 20 μm. (I and J) LCA RPE had decreased ciliation and cilia length, compared to controls. Values are mean ± 2 × SEM. n = 3 replicates of 50–100 cells. Statistical significance was determined using Student’s t test, n.s. = not significant. See also Figures S2 and S3.

Clonally derived iPSC lines were differentiated into RPE cells by growing them to confluence and withdrawing bFGF as described previously (Carr et al., 2009, Schwarz et al., 2015, Vugler et al., 2008). The *CEP290* mutation did not appear to delay or reduce the efficiency of the differentiation process, and pigmented colonies of RPE appeared at around week 4 in both LCA and control cell lines. Clusters of RPE were isolated manually after 8 weeks. Control and CEP290-LCA lines formed pigmented monolayers of RPE with typical polygonal morphology (Figure 2A) and were immunopositive for an array of RPE markers (Figures 2B–2E). Analysis of monolayer sections revealed polarized cells expressing MerTK apically and collagen IV in the basal layer (Figures 2D and 2E). RT-PCR confirmed the expression of the cryptic exon in LCA iPSC-RPE (Figure 2F). Interestingly, the ratio of the correctly spliced (26-27) to misspliced (26-X-27) transcript differed in the LCA RPE compared to LCA fibroblasts with the aberrant misspliced transcript accounting for only 50% of the total (Figure 2G). We examined the effect of *CEP290* missplicing on RPE cilia incidence and length and observed a slight reduction in LCA RPE cells compared to control (Figures 2H–2J).

### Differentiation of iPSCs to Opsin-Expressing Photoreceptors

**Figure 3:**
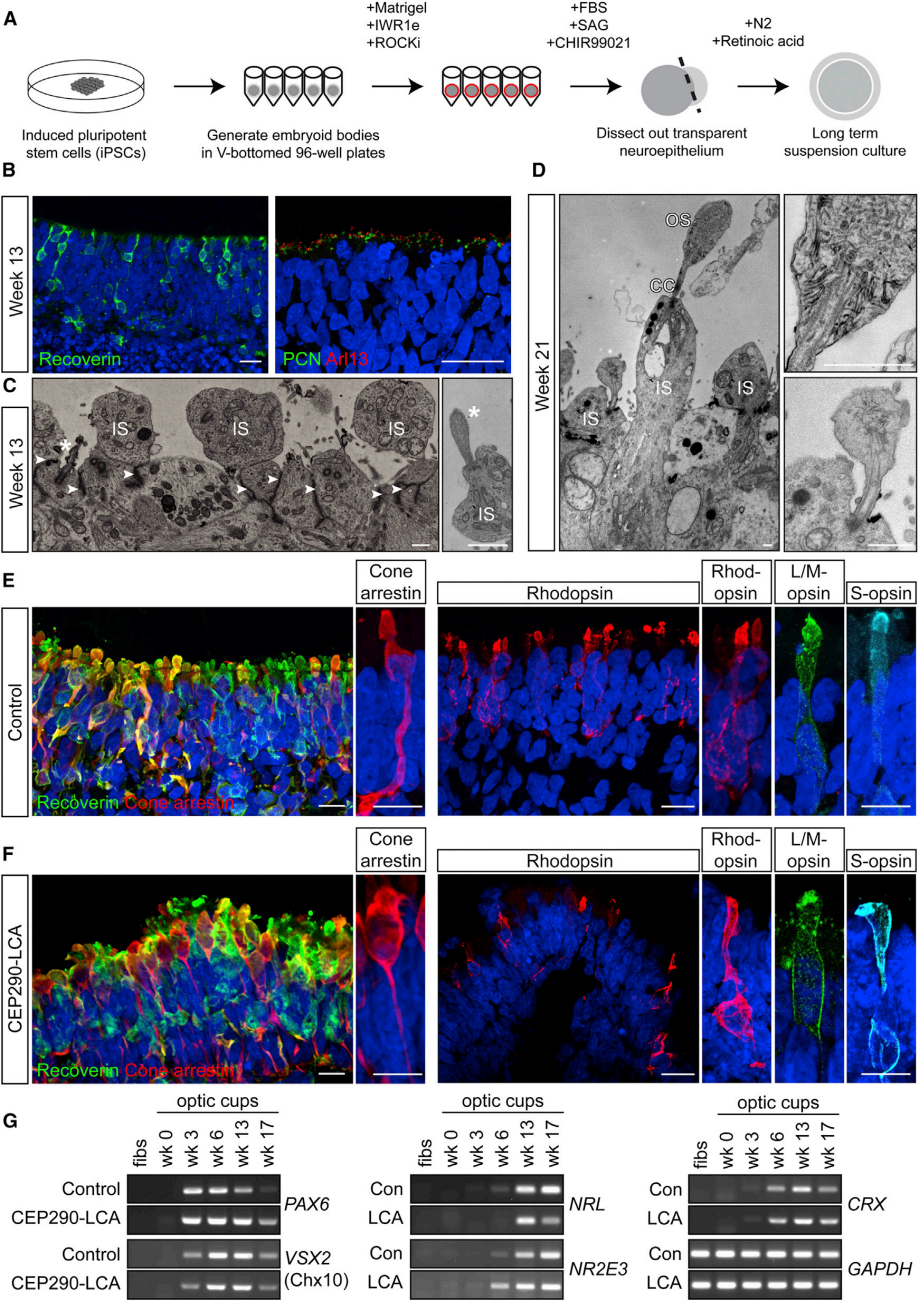
Generation of Opsin-Expressing Photoreceptors from iPSC Optic Cups following Long-Term 3D Suspension Culture (A) Schematic of differentiation process from iPSCs to photoreceptor cells based on previously described method for the generation of optic cups from human embryonic stem cells (Nakano et al., 2012). (B) In control optic cups, recoverin-positive cells formed an outer nuclear layer (ONL) at week 13. Cilia (Arl13, red; PCN, green) were aligned at the apical surface. Scale bar, 20 μm. (C) In control optic cups, electron micrographs (EMs) at the apical surface show the presence of cilia (^∗^), mitochondria-rich inner segments (IS), and tight junctions (arrowheads). Scale bar, 1 μm. (D) In control optic cups, EM shows the elongation of inner segments (IS), connecting cilium (CC), and the presence of developing outer segments (OS) at the ciliary tip. Inserts show higher-power images of developing OS disk stacks. Scale bar, 1 μm. (E and F) Control (E) and CEP290-LCA (F) optic cups were immunopositive for recoverin and cone arrestin (left panels), and rhodopsin and L/M and S cone opsin (right panels) at weeks 21 (E) and 17 (F). Scale bar, 20 μm (10 μm in zoom panel). (G) RT-PCR analysis of retinal development genes in control (Con) and CEP290-LCA fibroblasts (fibs) and optic cups at different times of development. See also Figure S4.

Photoreceptors are highly polar neurons with a distinctive inner and outer segment joined by a connecting cilium where CEP290 is thought to play an important role in protein trafficking. In order to study the effects of CEP290 depletion on photoreceptor genesis and maintenance, LCA and control iPSCs were differentiated using the 3D techniques for “optic cup” differentiation pioneered by Nakano et al. (Nakano et al., 2012). The progress of their differentiation was monitored by immunofluorescence and RT-PCR (Figure 3).

After 3–5 weeks of EB culture in retinal-cell-inducing medium, pouches of transparent neuroepithelium could be seen emerging from the embryoid bodies in both LCA and control cell lines. These pouches were dissected out and subcultured in retinal cell maturation media. After 2–3 further weeks in suspension culture, the pouches formed spherical aggregates with a thick, transparent mantel and histological features of an embryonic retina, including a Brn3/HuD-positive ganglion cell layer, a Chx10/Pax6-positive neuroblastic layer (Figure S4A), and occasional cone-arrestin- and recoverin-positive cells (Figure S4B). In the next stage of temporal development, the optic cups developed a more defined outer nuclear layer (ONL) containing migrating recoverin-positive progenitors (Figures 3B and S4B), as well as cone-arrestin-positive cells (Figure S4B), but no opsin expression (Figure S4C). As in normal development, the differentiation and organization of iPSCs into a polarized and stratified neuroepithelium was concomitant with polarization of the cilia at the apical layer of both control and LCA optic cups (Figure 3B). Electron microscopy (EM) showed apical tight junctions (arrow heads) and cilia (^∗^) emerging among mitochondria-rich inner segments (IS; Figures 3B and 3C).

At the latest time point investigated (week 21), EM analysis of control optic cups revealed the presence of multiple cilia and inner segments that had become elongated. The majority of the protruding cilia were broken at the distal tip, likely as a result of EM processing; however, occasionally disorganized but intact outer segments were observed elaborating from the connecting cilium (Figure 3C). Recoverin (rod, cone, and bipolar cells)- and cone-arrestin (cone specific)-positive cells were abundant across the apical layer of LCA and control optic cups (Figures 3D, 3E, and S4D). Rhodopsin-positive rod cells were detected across an ONL approximately five cell nuclei deep with the most intense staining seen in structures above the ONL (Figures 3D and 3E). L/M- and S-opsin-positive cone cells were also detected in LCA and control optic cups, but were less abundant than rods (Figures 3E and S4C). RT-PCR analysis of different developmental stages revealed concurrent expression of early differentiation genes such as *PAX6* and *VSX2/CHX10*, with similar parallel expression of cone-rod (*CRX*) and rod-specific genes (*NRL*, *NR2E3*) in both control and LCA optic cups (Figure 3F).

### LCA Optic Cups Have Severe *CEP290* Missplicing and Associated Ciliation Defects

**Figure 4:**
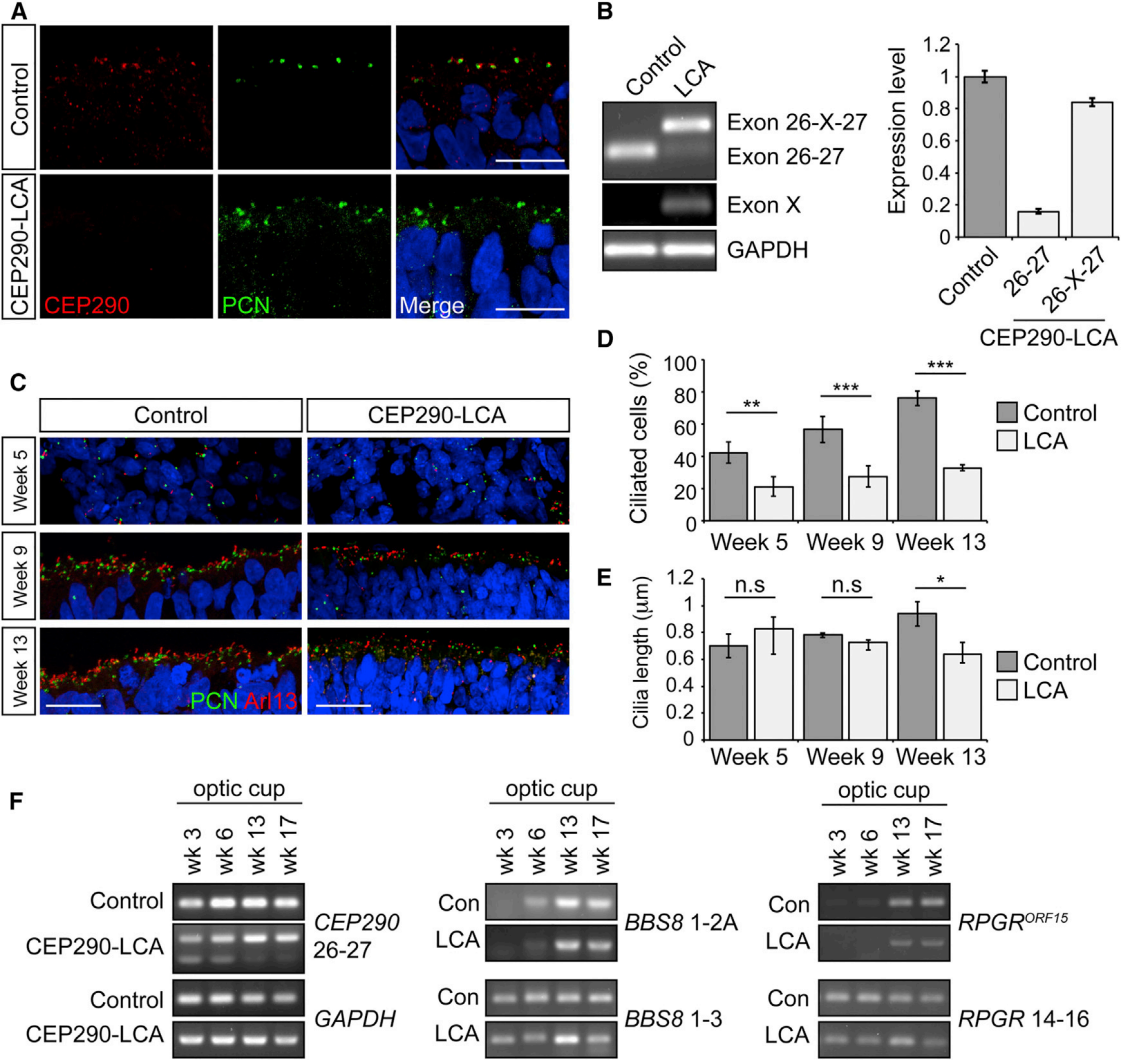
CEP290-LCA Optic Cups Lack CEP290 Expression and Have Decreased Ciliation (A) CEP290 (red) expression was absent from the basal body (pericentrin, PCN; green) in LCA optic cups at week 13, compared to controls. Scale bar, 10 μm. (B) RT-PCR analysis showed LCA optic cups had high relative levels of the cryptic exon. Values are mean ± 2 × SEM. n = 3. (C) Representative images of immunostaining of cilia (Arl13, red; PCN, green) in optic cups at weeks 5, 9, and 13. Scale bar, 20 μm. (D and E) Quantification of ciliation (D) and cilia length (E) in weeks 5, 9, and 13 optic cups, as determined by Arl13 and PCN staining. Values are mean ± 2 × SEM. n = 3 counts of 200 pericentrin-positive structures. Statistical significance was determined using one-way ANOVA with post hoc Tukey’s test, ^∗^p < 0.05, ^∗∗^p < 0.01, ^∗∗∗^p < 0.001, n.s. = not significant. (F) RT-PCR analysis of *CEP290* splicing and retinal-specific exons (*BBS8* 2A and *RPGR*^ORF15^) in control (Con) and CEP290-LCA optic cups at different times of development. See also Figure S5.

In control optic cup photoreceptors, CEP290 colocalized with pericentrin at the base of the connecting cilia, whereas in LCA optic cups, CEP290 was not detectable at the connecting cilia (Figures 4A and S5A). RT-PCR confirmed the presence of the cryptic exon in LCA optic cups (Figures 4B and S5A). Strikingly, very little correctly spliced *CEP290* transcript was expressed in LCA optic cups (10%–20% of total CEP290 mRNA), whereas in LCA fibroblasts and RPE, it was 40% and 50%, respectively (Figures 1B, 2F, and 4B). Ciliation in developing optic cups was examined in LCA and control cells. Prior to neuroepithelial differentiation, cilia were randomly dispersed in the center of the embryoid bodies. As the EBs underwent neuroepithelial differentiation, extensive migration and polarization of cilia to the apical surface occurred in both control and LCA optic cups (Figures 4C and S5C). Quantification of ciliated basal bodies in developing optic cups revealed an increase in ciliogenesis with time and a significant reduction in cilia incidence in the LCA cells at all stages of optic cup development (Figures 4D and S5D). Cilia length was significantly reduced at the latest time point only, correlating with the very low levels of full-length *CEP290* transcript (Figures 4E, 4F, and S5E). The missplicing of *CEP290* was examined via RT-PCR during the differentiation period of the optic cups (Figure 4F). At week 3 the splicing of *CEP290* was broadly similar to that seen in LCA fibroblasts, iPSCs, and RPE. During subsequent development, the amount of 26-X-27 increased and 26-27 decreased. This correlated with expression of retina-specific isoforms of *BBS8* (Murphy et al., 2015) and *RPGR* (Kirschner et al., 1999) (Figure 4F), suggesting that increased missplicing of *CEP290* is associated with photoreceptor differentiation and the inclusion of photoreceptor-specific exons.

### Antisense Morpholino Treatment Reduces Aberrant Splicing and Restores Ciliogenesis in CEP290-LCA RPE and Photoreceptors

**Figure 5:**
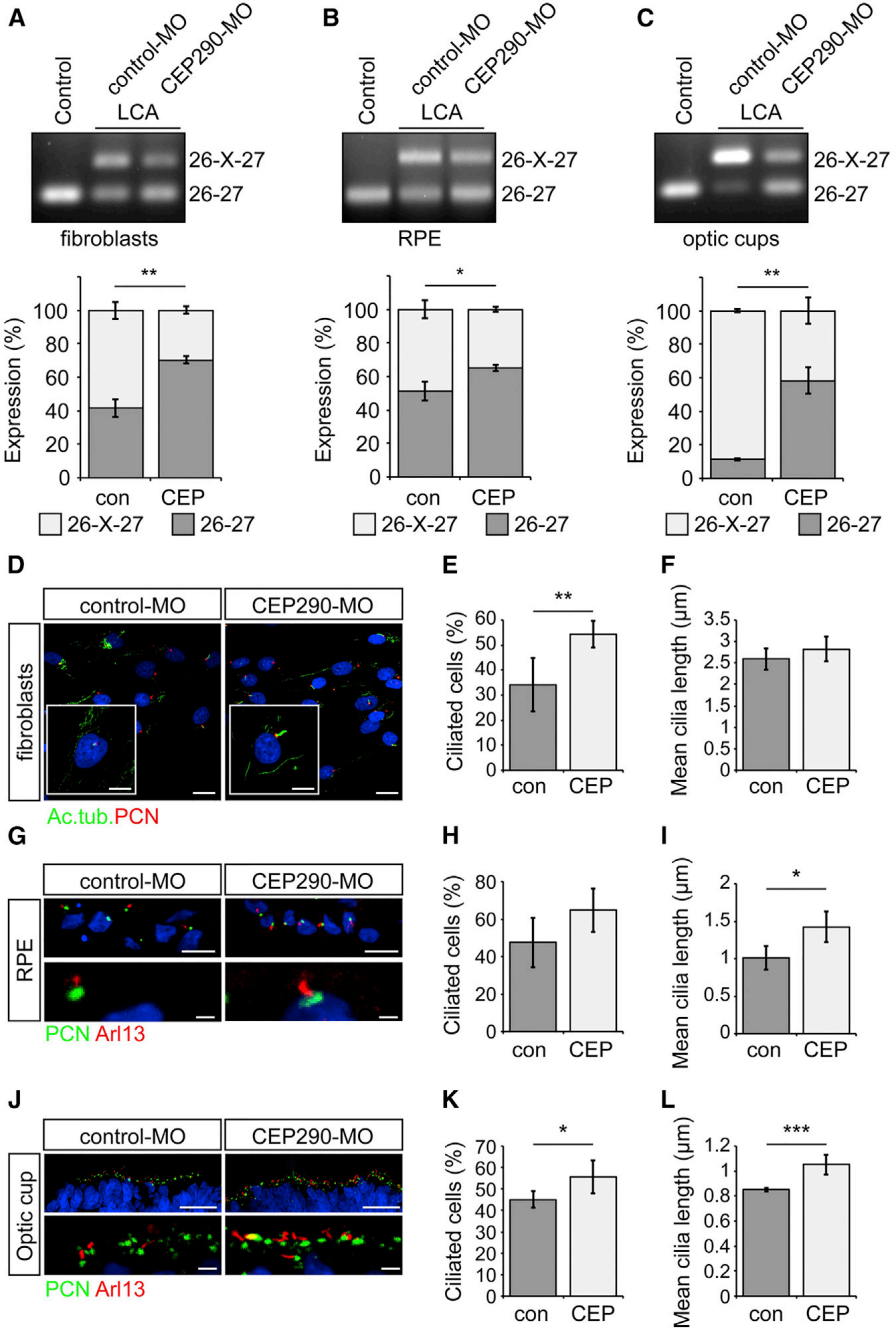
CEP290-MO Treatment Increases Wild-Type *CEP290* Transcript Levels, Reduces Cryptic Exon Expression, and Rescues Ciliation (A–C) RT-PCR analyses of *CEP290* exon 26-27 and quantification of bands in LCA cells: (A) fibroblasts, (B) RPE, and (C) optic cups. Values are mean ± 2 × SEM. Statistical significance was determined using Student’s t test, ^∗^p < 0.05, ^∗∗^p < 0.01. n = 3 for each cell type. (D–L) Representative images of cilia (Ac. tub., green; PCN, red; or Arl13, red; PCN, green) and quantification of cilia incidence and length in patient cells: (D–F) fibroblasts, (G–I) RPE, and (J–L) optic cups. Values are mean ± 2 × SEM. Statistical significance was determined using Student’s t test, ^∗^p < 0.05, ^∗∗^p < 0.01, ^∗∗∗^p < 0.001. (E and F) n = 4 counts of at least 80 cells per treatment, (H and I) n = 3 counts of at least 70 cells per treatment, and (K and L) n = 5 counts of at least 400 pericentrin-positive structures. (D–F) Scale bar, 20 μm (10 μm in zoom panel); (G–I) scale bar, 10 μm (1 μm in zoom panel); and (J–L) scale bar, 20 μm (1 μm in zoom panel). See also Figures S4 and S6.

AONs designed to target the aberrant splice donor site in *CEP290* have been shown to increase the relative proportion of correctly spliced *CEP290* and restore ciliation in LCA fibroblasts (Collin et al., 2012, Gerard et al., 2012). Assessing the effect of AONs on CEP290-LCA RPE and photoreceptors provides a unique opportunity to assess their therapeutic efficacy in the tissue that manifests LCA disease pathology. A 25 bp antisense morpholino (CEP290-MO) was developed against the c.2991+1665A>G change in *CEP290* and tested on LCA fibroblasts alongside a standard control-MO (Figure S6). RT-PCR revealed that an increase in *CEP290* 26-27 transcript and concurrent reduction in cryptic exon inclusion occurred after 48 hr at doses of 10 μM and above (Figure S6A). At 10 μM, levels of correctly spliced *CEP290* mRNA increased by ∼25%, compared to control-MO (Figures 5A and S6D). MO treatment was still effective 9 days after CEP290-MO treatment and increased the levels of correctly spliced product to over 80% of the total transcript (Figure S6E). Control and LCA RPE were treated with control- or CEP290-MO once every 7 days for 14 days. Similar to the LCA fibroblasts, the treatment led to an increase in correctly spliced transcript in RPE cells, but the relative increase was reduced compared to fibroblasts (15%), probably because of the higher steady-state levels of the correctly spliced exon 26-27 transcript in LCA RPE cells (Figure 5B).

Depletion of CEP290 has been reported to affect photoreceptor viability soon after photoreceptor differentiation (Chang et al., 2006). Therefore, we treated LCA optic cups at week 13, a point in their temporal development when recoverin- and cone-arrestin-positive photoreceptor progenitors were observed and aberrant 26-X-27 splicing was high. At this time point, ciliation was significantly reduced in LCA optic cups relative to controls, but full photoreceptor differentiation had not occurred (Figures S4B, 4C, and 4D). Optic cups were treated with control- or CEP290-MO every 3–4 days for 4 weeks. Unlike RPE and fibroblasts, the 3D optic cups comprise several cell layers tightly packed together (Figures 3D and S4). In order to assess the ability of the MO to penetrate the optic cups, the localization of a fluorescein-tagged control-MO was determined using live cell confocal microscopy. Both punctate and dispersed fluorescein could be observed throughout all cell layers 48 hr posttreatment (Figure S4E). CEP290-MO-treated LCA optic cups had significantly increased levels of the correctly spliced exon 26-27 transcript, increasing to over 50% of the total transcript in LCA line 1 (Figure 5C) and up to 70% in LCA line 2 (Figures S7A and S7B). This increase was considerably higher than the CEP290-MO-induced changes observed in fibroblasts and RPE cells, implying that the MO therapy for CEP290 is particularly potent in photoreceptors, most likely because of the higher basal level of aberrant splicing observed in these cells.

CEP290-MO treatment also had a functional impact on ciliation levels, as measured by incidence of Arl13 staining, in all cell types investigated. In LCA fibroblasts, cilia incidence was significantly increased, but cilia length remained similar (Figures 5D–5F). In LCA RPE cells (Figures 5G–5I), both cilia incidence and length were increased, but only the increase in length reached statistical significance. In LCA optic cups, CEP290-MO treatment significantly increased both the number and length of cilia emerging from recoverin- and cone-arrestin-positive photoreceptor progenitors, in both cell lines (Figures 5J–5L and S7C–S7E).

### CEP290-MO Therapy Restores Defective Cilia Protein Traffic in LCA Fibroblasts and Optic Cups

**Figure 6:**
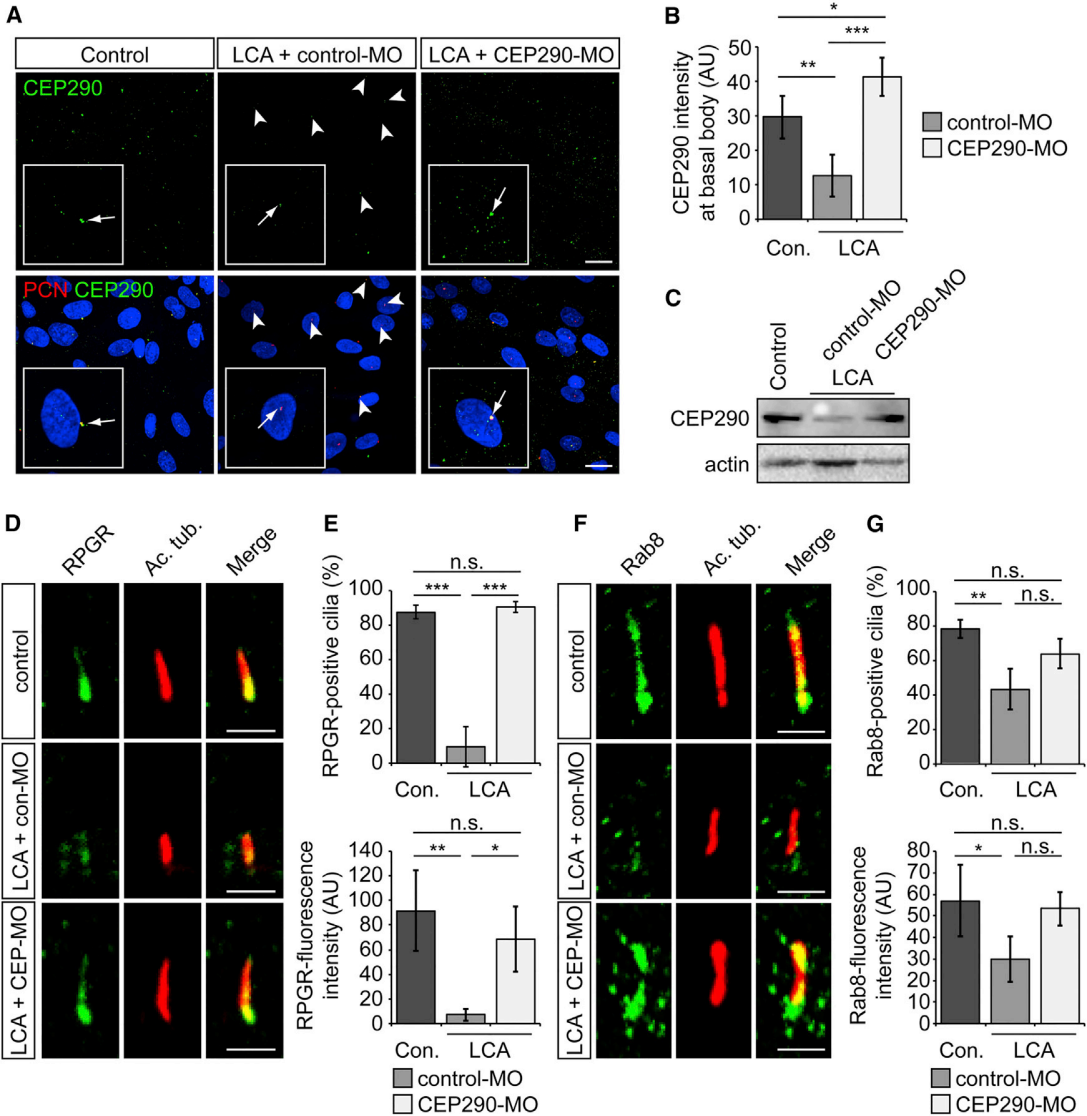
CEP290-MO Rescues CEP290 Protein and the Cilia Traffic of RPGR and Rab8 in LCA Fibroblasts (A) Representative images of control and LCA fibroblasts treated with control- or CEP290-MO and stained with CEP290 (green) and PCN (red). Arrowheads show drastically reduced CEP290 basal body localization in LCA fibroblasts compared. (B) Quantification of CEP290 immunofluorescence intensity at the basal body. Values are mean ± 2 × SEM. n = 3 replicates of at least 30 cells. Statistical significance was determined using one-way ANOVA with post hoc Tukey’s test, ^∗^p < 0.05, ^∗∗^p < 0.01, ^∗∗∗^p < 0.001. (C) Western blot showing increased level of CEP290 protein in LCA fibroblasts after CEP290-MO treatment. (D and F) Representative images of (D) RPGR or (F) Rab8 (green) localization in the cilium in control or LCA fibroblasts. Ciliary axoneme is marked by acetylated α-tubulin (Ac. tub.; red). (E and G) Quantification of (E) RPGR or (G) Rab8 localization at the base of the cilia (upper) and fluorescence intensity in the axoneme (lower). Values are mean ± 2 × SEM. n = 3 replicates of 50 cells. Statistical significance was determined using one-way ANOVA with post hoc Tukey’s test, ^∗^p < 0.05, ^∗∗^p < 0.01, ^∗∗∗^p < 0.001, n.s. = not significant. Scale bar, 2 μm. See also Figure S6.

The ability of CEP290-MO treatment to restore functional CEP290 protein expression was investigated in LCA fibroblasts. CEP290 was almost undetectable at the basal body by immunofluorescence, whereas following CEP290-MO treatment it could be detected at levels similar to control (Figures 6A and 6B). Western blotting revealed an increase in CEP290 protein levels (Figure 6C). To determine whether the restored protein level had an effect on known CEP290-interacting proteins, we investigated the traffic of RPGR and Rab8 to cilia in LCA fibroblasts treated with control- or CEP290-MO. In control-MO treated cells, the ciliary targeting of RPGR and Rab8 was disrupted and both proteins had impaired ciliary localization, compared to control fibroblasts (Figures 6D–6G). Importantly, the traffic of these proteins to cilia was significantly rescued by CEP290-MO treatment (Figures 6E and 6G).

**Figure 7:**
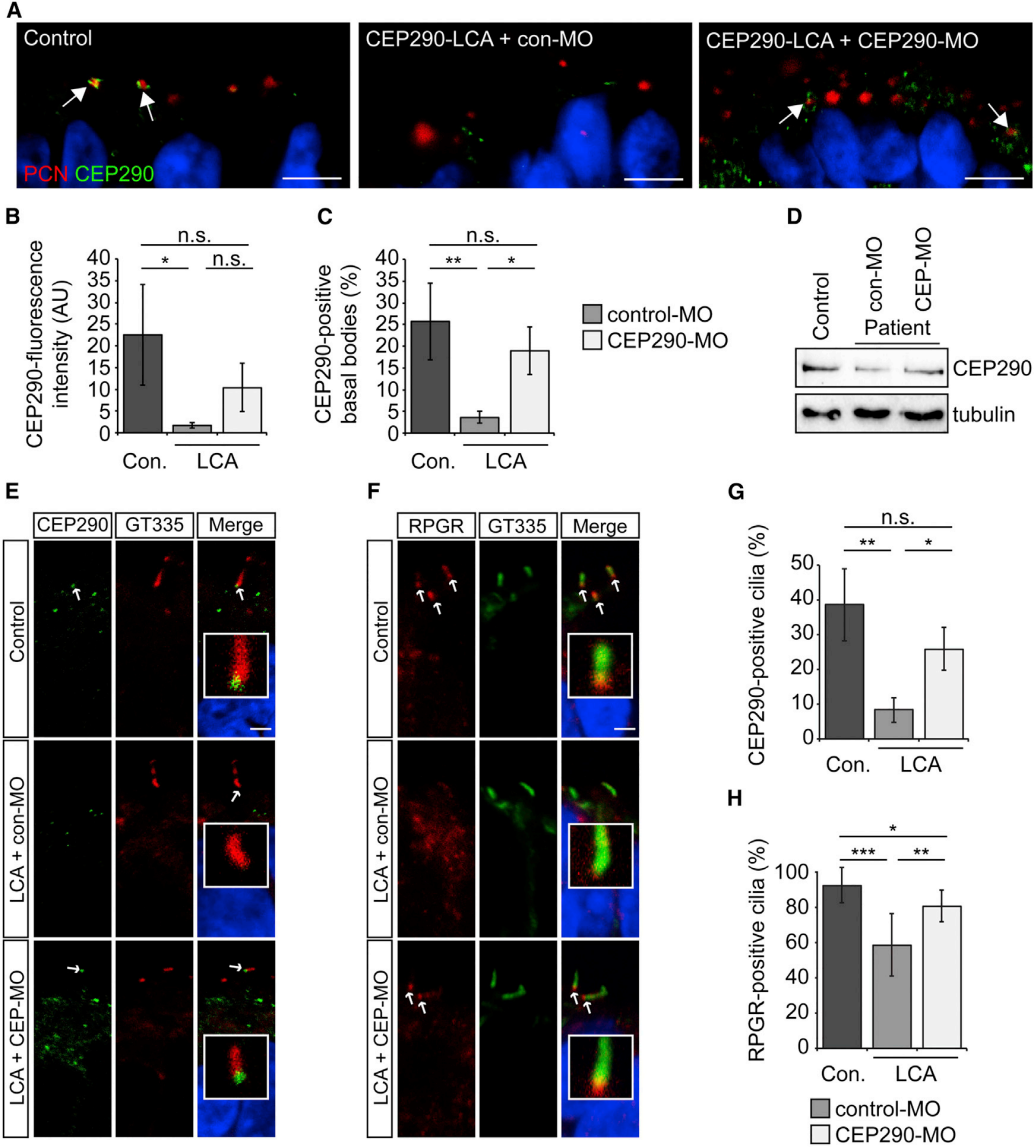
CEP290-MO Rescues CEP290 Protein and the Cilia Traffic of RPGR in LCA Optic Cups (A) Representative images of control and LCA optic cups treated with control- or CEP290-MO and stained with CEP290 (green) and PCN (red). Arrows show overlap of CEP290 with PCN in control- and CEP290-MO-treated LCA optic cups, respectively. Scale bar, 5 μm. (B and C) Quantification of (B) CEP290 immunofluorescence intensity and (C) CEP290 localization at the basal body. Values are mean ± 2 × SEM. n = 3 replicates of at least 45 cilia. Statistical significance was determined using one-way ANOVA with post hoc Tukey’s test, ^∗^p < 0.05, ^∗∗^p < 0.01, n.s. = not significant. (D) Western blot showing increased level of CEP290 protein in LCA optic cups after CEP290-MO treatment. (E and F) Representative images of (E) CEP290 or (F) RPGR (green) localization at the connecting cilium (CC) in control or LCA optic cups. Ciliary axoneme is marked by polyglutamylated tubulin (GT335; red). (G and H) Quantification of (G) CEP290 or (H) RPGR localization at the CC. Values are mean ± 2 × SEM. n = 3 replicates of at least 50 cilia. Statistical significance was determined using one-way ANOVA with post hoc Tukey’s test, ^∗^p < 0.05, ^∗∗^p < 0.01, ^∗∗∗^p < 0.001, n.s. = not significant. Scale bar, 2 μm. See also Figure S7.

We next investigated the ability of CEP290-MO to restore CEP290 protein levels and ciliary localization of RPGR in optic cups. CEP290-MO treatment significantly increased the number of CEP290-positive cilia at the basal body and transition zone (Figures 7A–7C, 7E, 7G, S7F, and S7G), as well as increasing CEP290 protein levels detected by western blotting (Figure 7D). In control optic cups, RPGR was detected by immunofluorescence at the ciliary transition zone, whereas in LCA optic cups there was a significant reduction in detectable RPGR, indicating that depletion of CEP290 reduces its ciliary localization in LCA photoreceptor progenitors. Critically, CEP290-MO treatment significantly rescued RPGR targeting to the cilium (Figures 7F and 7H).

## Discussion

In this study, we have used LCA patient-derived cells to examine the cellular consequences of reduction of CEP290 expression, particularly in the context of retinal cells, and tested a potential therapy for alleviating the effect of reduced levels of CEP290. We characterized fibroblasts, iPSCs, iPSC-derived RPE, and optic cups from a patient homozygous for the common *CEP290* c.2991+1665A>G mutation. We verified that all patient-derived cell types have reduced CEP290 expression, at both mRNA and protein expression levels, compared to control cells. The reduction in CEP290 expression in patient-derived cells reduced ciliation. Importantly, full-length CEP290 is not completely lost in patient-derived cells, suggesting residual expression is sufficient for ciliogenesis to take place in the majority of ciliated tissues where CEP290 is expressed, with the exception of the retina.

Mutations in *CEP290* cause other partially overlapping, but distinct, ciliopathies such as JBTS, MKS, NPHP, or SLSN. The pleiotropic manifestation of *CEP290* disease has eluded explanation; recently, it has been suggested that it may be a consequence of the combination of mutation position and severity, with exon skipping of some premature stop codons (Drivas et al., 2015). Nevertheless, the observations of 40%–50% of full-length *CEP290* transcript and protein in *CEP290* c.2991+1665A>G fibroblasts (den Hollander et al., 2006, Drivas et al., 2015) are hard to reconcile with the absence of disease phenotype in carriers of null alleles. Here, we provide direct evidence that tissue-specific differences in splicing are also likely to contribute to the genotype:phenotype relationship. The *CEP290* c.2991+1665A>G allele is only associated with nonsyndromic retinal disease, and our data suggest that this is likely to be a result of higher levels of aberrant splicing in the human retina. Therefore, this intronic change results in photoreceptors having a greater deficit of CEP290 protein than other cells, leading to the disease affecting them preferentially, as opposed to a greater vulnerability of photoreceptors to a similar reduction in CEP290 function.

The reasons for this different mRNA processing are currently unclear, but it was recently shown that the human retina has unexpectedly high levels of splicing diversity (Farkas et al., 2013). Furthermore, the mutation of ubiquitously expressed and highly conserved splicing factors *PRPF6*, *PRPF8*, and *PRPF31* causes retinal degeneration (Wright et al., 2010), suggesting a specialized role of the splicing machinery in the retina. Indeed, these splicing factors appear to be recruited to the base of primary cilia and are involved in cilia function (Wheway et al., 2015), suggesting a potential feedback loop between cilia function and mRNA processing. Interestingly, two naturally occurring animal models of *CEP290* retinopathy, the *rd16* mouse and Abyssinian cat (Chang et al., 2006, Menotti-Raymond et al., 2007), are also caused by different aberrant splicing events, and a deep intronic mutation in *OFD1* results in partial cryptic exon missplicing and causes a form of X-linked retinitis pigmentosa, as opposed to male lethality caused by null alleles (Webb et al., 2012). The increase in aberrant splicing in optic cups correlated with photoreceptor differentiation and the inclusion of photoreceptor-specific exons in other genes. Therefore, the retina appears to have a highly tuned RNA splicing machinery that potentially exposes it to the risk of aberrant exon splicing more than other tissues.

The availability of CEP290 model organisms, from *Chlamydomonas* to cat, has facilitated the understanding of CEP290 function (Chang et al., 2006, Craige et al., 2010, Menotti-Raymond et al., 2007). CEP290 is known to be important in ciliogenesis and the formation of the Y-shaped linkers that connect the microtubule bundles to the membrane in the transition zone, suggesting an important role of CEP290 at the ciliary gate, which regulates intraflagellar transport components and entry into the cilium (Craige et al., 2010, Reiter et al., 2012). We have studied a range of human cells at a previously uninvestigated level of detail. Our data show that reductions of CEP290 by 50%–60% result in detectable cilia defects; however, this does not affect the ability of iPSCs to differentiate to a variety of cell types, suggesting there is sufficient CEP290 to facilitate a level of cilia function that is consistent with the signaling involved in cell differentiation. This is consistent with the retinal-only phenotype observed in CEP290-LCA patients. Furthermore, within the retina cell types our data show that the RPE are relatively unaffected, with relatively high levels of wild-type *CEP290* transcript. This confirms that the photoreceptors are the primary site of pathology, as observed in the *rd16* mouse model.

Studies in human fibroblasts have suggested AON therapy may be able to combat *CEP290*-associated aberrant splicing (Collin et al., 2012, Gerard et al., 2012); however, testing these potential therapies in animals has not been possible because the mouse and cat models have different causative mutations and the therapy is sequence specific. An attempt to produce a humanized mouse model of CEP290-LCA containing human exons 26 and 27, and intron 27 with or without the splicing mutation, resulted in the creation of a separate cryptic exon, highlighting the difficulty in studying human splicing mutations in animal models (Garanto et al., 2013). Therefore, modeling the disease in a human sequence-specific context by studying iPSC-derived photoreceptors presents an ideal solution. *CEP290*-specific MO treatment restored normal splicing and led to increased CEP290 protein production that was sufficient to rescue ciliation and recruitment of Rab8 and RPGR to fibroblast cilia. Importantly, CEP290-MO also increased CEP290 protein, restored ciliation, and significantly improved the recruitment of the important photoreceptor protein, RPGR, to the connecting cilia of photoreceptor progenitor cells within optic cups. This clearly demonstrates significant recovery of cilia traffic in LCA photoreceptors, which may be critical to photoreceptor function and viability in vivo.

Optical coherence tomography (OCT) analysis has shown that CEP290-LCA patients often retain central retina structure and that photoreceptor ONL thickness is normal in the cone-rich fovea, but severely reduced toward the rod-rich peripheral retina (Boye et al., 2014, Cideciyan et al., 2007, Pasadhika et al., 2010). Indeed, our *CEP290* patient has central photoreceptor preservation at over 40 years of age (Figure S1). This suggests that the therapeutic window for CEP290-LCA caused by this intronic change might be longer than previously estimated and that photoreceptors might be more amenable to gene-directed therapies aimed at correcting splicing such as that described here, especially given that *CEP290* is too big for conventional AAV gene therapy (Allocca et al., 2008). Lentivirus has proven an effective vector system for large cDNAs such as *CEP290* (Burnight et al., 2014). However, the observed cytotoxicity following CEP290 overexpression in cultured fibroblasts suggests that significant improvements will be required to translate this approach into a safe therapy for LCA. A recent study has shown that intravitreal injection of oligonucleotides can alter photoreceptor splicing (Gérard et al., 2015) and is relatively safe. Vitravene, which is used to treat CMV retinitis, was the first antisense therapeutic approved by the FDA (Marwick, 1998), and modified oligonucleotides might be stable in the vitreous with good accessibility to the photoreceptor cell layer for several months (Murray et al., 2015). Our data suggest that AON therapy, either as intravitreal injection or through viral delivery, could be a practical therapeutic option for treating CEP290-LCA and restoring gene function. Furthermore, this study highlights the value of using iPSC-derived cells to study photoreceptor development and function, interrogate disease mechanisms, and test potential therapies for human mutations in a genomic context.

## Experimental Procedures

### Reprogramming of Fibroblasts to iPSCs

Following informed consent, a skin biopsy was obtained from a male *CEP290* individual. Control BJ and LP6 fibroblasts and iPSCs were produced and characterized as previously described (Schwarz et al., 2015). The study followed the tenets of the Declaration of Helsinki and was approved by the Moorfields and Whittington Hospitals’ local Research Ethics Committees and the NRES Committee London Riverside Ethics Committee (REC 12/LO/0489). Fibroblasts were cultured from the skin biopsy as previously described (Schwarz et al., 2015). iPSCs were generated from fibroblasts using the following integration-free episomal plasmids from Addgene: pCXLE-hOCT3/4-shp53-F, pCXLE-hUL, and pCXLE-hSK. Reprogramming protocol was as previously described (Okita et al., 2011, Schwarz et al., 2015). In total, 1 × 10^6^ cells were mixed with 1 μg of each plasmid in 100 μl Nucleofector solution from a Cell Line Nucleofector kit (Lonza) before electroporation via Amaxa Nucleofector I device. Electroporated cells were plated on 0.2% gelatin-coated 10 cm^2^ plates and cultured in fibroblast media (DMEM supplemented with 10% fetal bovine serum [FBS], 1 mM nonessential amino acids [NEAA], 1 mM GlutaMax, and 1% penicillin/streptomycin [P/S; Life Technologies]) supplemented with 0.5 mM sodium butyrate. After 7 days the cells were replated at 2 × 10^5^ cells per well into a Matrigel (Corning)-coated six-well plate. The next day, the media was changed to mTESR-E8 media (Stem Cell Technologies) until iPSC colonies appeared (approximately 25 days). Clonal iPSC lines were mechanically isolated and maintained in mTESR-E8.

### Differentiation of iPSCs to RPE and Optic Cups

For spontaneous differentiation of iPSCs into RPE, iPSCs were passaged into T25 cell culture flasks and maintained in mTESR-E8 media for 10 days, before the media was exchanged for RPE differentiation media (KnockOut DMEM supplemented with 20% KnockOut Serum Replacement [KOSR], 1 mM NEAA, 1 mM GlutaMax [all Life Technologies], and 100 μM β-mercaptoethanol) until pigmented colonies were formed. iPSC-RPE colonies were manually isolated using crescent blades as previously described (Schwarz et al., 2015) before being plated at a density of 5 × 10^4^ cells per cm^2^ in X-VIVO-10 media (Scientific Laboratory Supplies). The media was changed every 3–4 days until a pigmented monolayer had formed (approximately 6–8 weeks).

Directed differentiation of iPSCs into 3D optic cups was based on the protocol by Nakano et al. (Nakano et al., 2012). iPSCs were dissociated using TrypLE (Life Technologies) and plated at a density of 9,000 per well in V-shaped 96-well plates in EB media (GMEM supplemented with 20% KOSR, 1 mM NEAA, 1 mM GlutaMax, 1 mM sodium pyruvate, and 100 μM β-mercaptoethanol) supplemented with 20 μM Y-27632 (Millipore) and 3 μM IWR1e (Calbiochem). After 48 hr (day 2) cells were topped up with EB media containing 20 μM Y-27632, 3 μM IWR1e, and 2% Matrigel (EB2 media). EB2 media was exchanged every 2 days until day 12 when the EBs were transferred to 25-well nonadherent dishes for further culture in EB media with 10% FBS, 1% Matrigel, 20 μM Y-27632, and 100 nM smoothened agonist (SAG; Enzo Life Sciences). After 3 days (day 15) media was exchanged for EB media with 10% FBS, 1% Matrigel, 20 μM Y-27632, 100 nM SAG, and 3 μM CHIR99021 (Tocris). Media was exchanged every 2 days until day 18 when EB media was exchanged for neural retinal differentiation (NR) media (DMEM-F12 supplemented with 10% FBS, 1× N2 supplement [Life Technologies], and 0.5 μM retinoic acid [RA; Tocris]). Pouches of transparent neuroepithelium were manually isolated under a dissecting microscope at day 30 and transferred to fresh 25-well nonadherent dishes. These were maintained for up to 21 weeks, changing NR media every 3–4 days. Please see Supplemental Experimental Procedures for details of RNA extraction and RT-PCR, electron microscopy, immunoblotting, immunofluorescence, and imaging of cells.

### Morpholino Treatment

CEP290-specific antisense MO oligonucleotide was purchased from GeneTools, with the sequence 5′-GGATAGGTATGAGATACTCACAATT-3′. Standard control MO (5′- CCTCTTACCTCAGTTACAATTTATA-3′) was also from GeneTools. MOs were diluted from a stock concentration of 1 mM to concentrations indicated in the text, in appropriate sterile culture media supplemented with 6 μM EndoPorter (GeneTools).

## Author Contributions

D.A.P. and A.L. designed and performed experiments, analyzed and interpreted data, prepared figures, and wrote the manuscript. C.M.R. designed and performed experiments, and analyzed and interpreted data. A.-J.F.C., K.J., N.S., and N.K. performed experiments and analyzed data. P.M.M. performed the EM. M.N.M. and L.dC. prepared ethics application and obtained ethics committee approval, and M.N.M performed the patient skin biopsy. S.H. clinically assessed the patient. L.dC. and A.J.H. analyzed and interpreted data. J.-M.G., A.T.M., and P.J.C. conceived the study, and analyzed and interpreted data. M.E.C. conceived and supervised the study, designed experiments, interpreted data, and wrote the manuscript. All authors contributed to editing the manuscript.

## Supplemental Information

Document S1. Supplemental Experimental Procedures and Figures S1–S7

Document S2. Article plus Supplemental Information
