# Supplementary material for: Identification and Correction of Mechanisms Underlying Inherited Blindness in Human iPSC-Derived Optic Cups
Source: Cell Stem Cell. 2016 Jun 2;18(6):769–81. doi: 10.1016/j.stem.2016.03.021 (PMC4899423; doi:10.1016/j.stem.2016.03.021)
Supplement: Document S1. Supplemental Experimental Procedures and Figures S1–S7 [file mmc1.pdf]

Cell Stem Cell, Volume *18*

## Supplemental Information

# Identification and Correction of Mechanisms Underlying Inherited Blindness in Human iPSC-Derived Optic Cups

**David A. Parfitt, Amelia Lane, Conor M. Ramsden, Amanda-Jayne F. Carr, Peter M. Munro, Katarina Jovanovic, Nele Schwarz, Naheed Kanuga, Manickam N. Muthiah, Sarah Hull, Jean-Marc Gallo, Lyndon da Cruz, Anthony T. Moore, Alison J. Hardcastle, Peter J. Coffey, and Michael E. Cheetham**

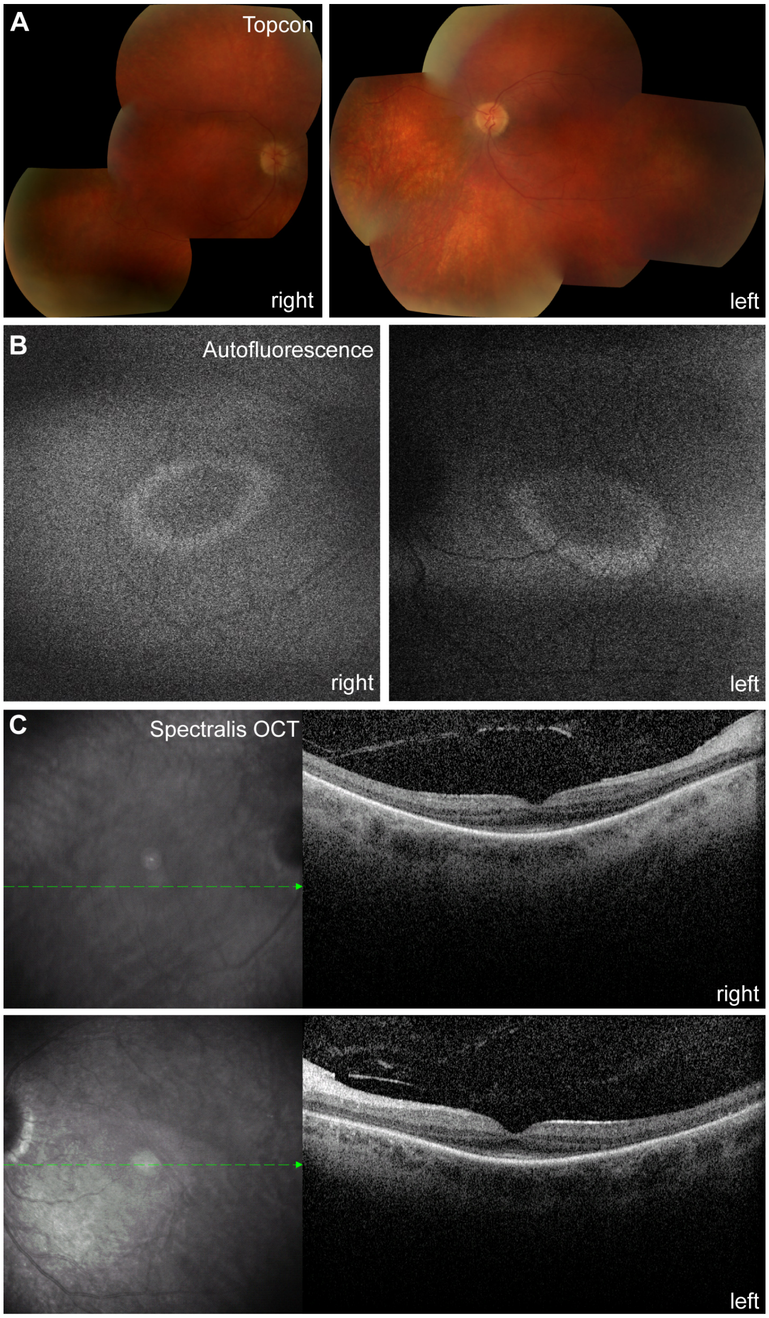


**Figure S1. Related to Figure 1. CEP290 patient retinal imaging**. (**A**) Topcon composite fundus images taken at age 42. (**B**) Autofluorescence imaging showing a ring of hyperfluorescence in the central retina surrounding the macula taken, at age 39. (**C**) Spectralis OCT imaging at age 39. Fundus image on the left, the green line shows the position of the cross section shown on the right panel. Note central perifoveal area of preserved ONL.

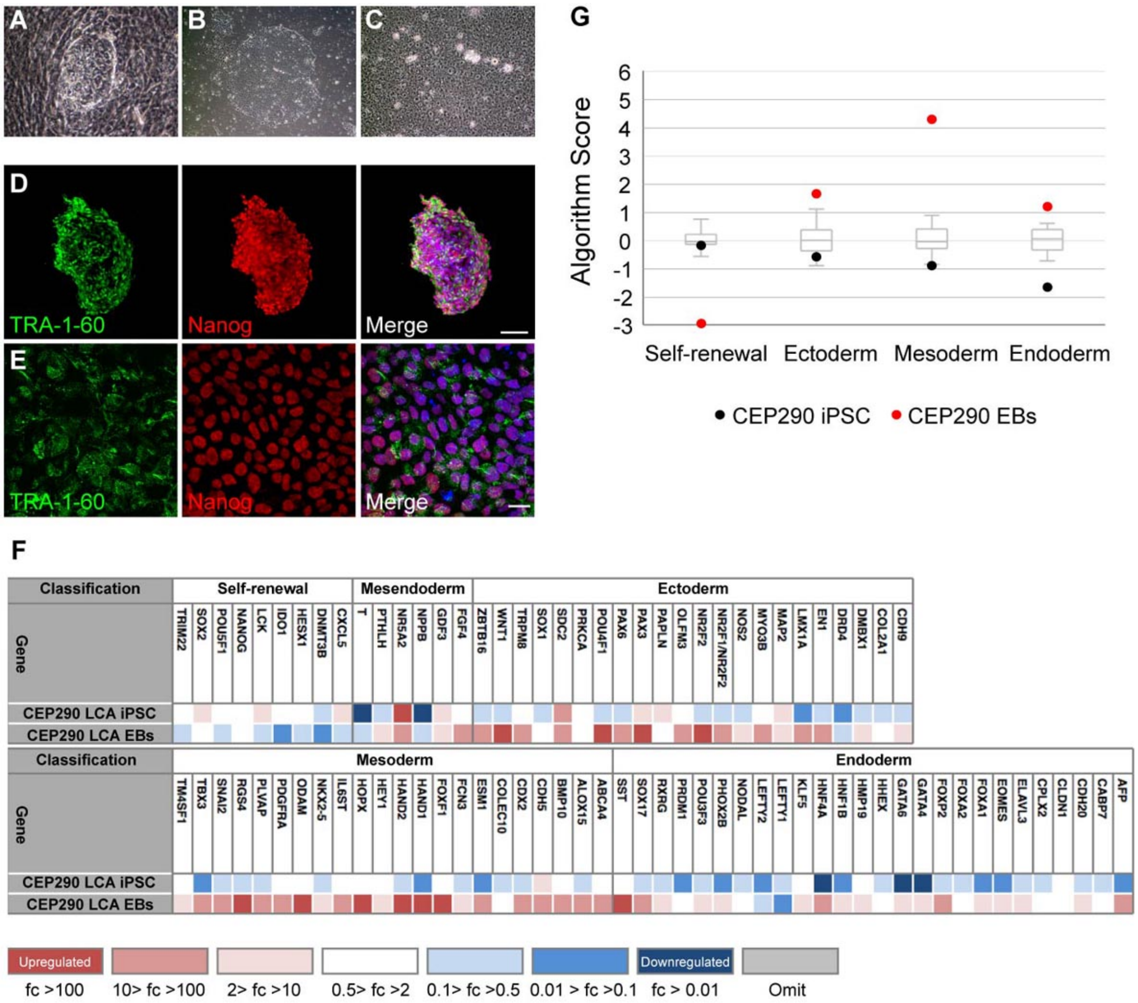


**Figure S2. Related to Figure 2. CEP290 LCA iPSC characterisation.** (**A**, **B**) iPSC colonies appeared approximately 30 days post-electroporation and were excised and replated. (**C**) iPSCs showed typical morphology and (**D**, **E**) expression of pluripotency markers TRA-1-60 (green) and Nanog (red). Scale bar = 100µm in (**D**) and 20µm in (**E**). (**F**) Heat map showing expression of key embryonic stem cell and germ layer markers in iPSC and following 7 days of undirected embryoid body differentiation using the TaqMan hPSC Scorecard assay. Colours correspond to relative up- or down-regulation of genes compared to a reference set taken from 12 embryonic stem cell and 12 iPSC lines. (**G**) Box and whisker plot showing the average expression of self-renewal and embryonic germ layer genes in the ScoreCard reference set stem cell lines. CEP290 LCA iPSC (black dots) fall within the expected range in terms of self-renewal, ectoderm and mesoderm gene expression. Expression is significantly upregulated in all three germ layers following undirected embryoid body differentiation (red dots) and significantly downregulated in self-renewal genes.

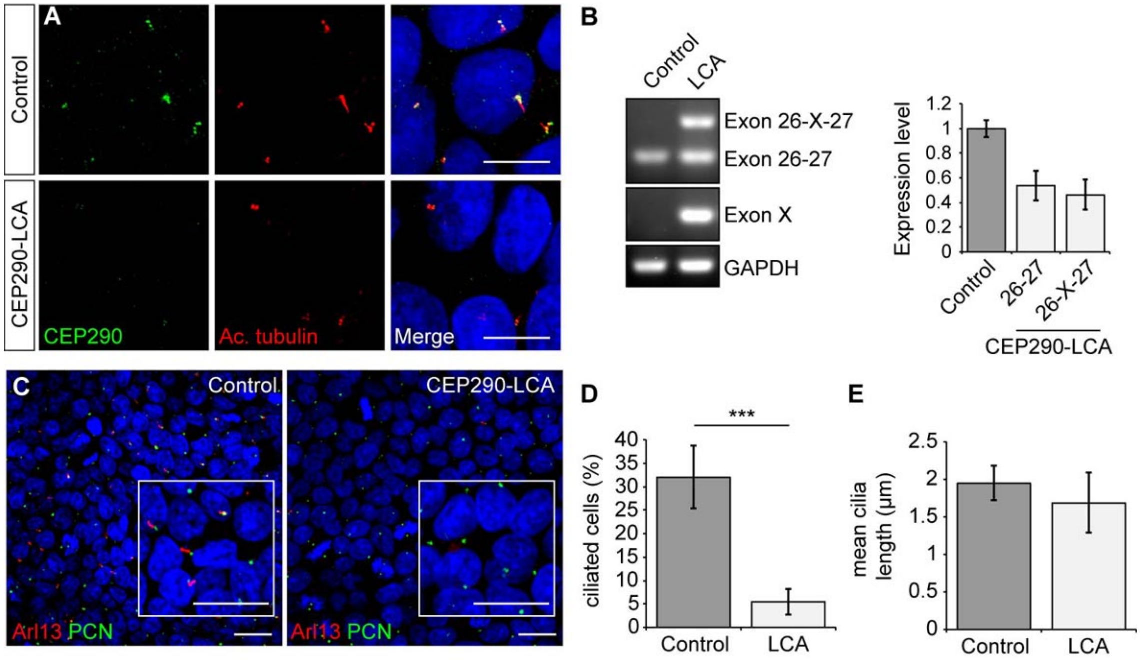


**Figure S3. Related to Figure 2. CEP290-LCA iPSC aberrant splicing and cilia defects.** (**A**) Representative images of CEP290 (green) expression in iPSCs. Acetylated a-tubulin (red) was used to mark the ciliary axoneme. Scale bar = 10μm. (**B**) CEP290 LCA iPSCs showed expression of cryptic exon via RT-PCR analysis using primers to exons 26-27 and exon X, as described. (**C**) Representative images of cilia (axenome = Arl13; red and basal body = pericentrin (PCN); green) in iPSCs. Scale bar = 20μm. Quantification of iPSC ciliation (**D**) and cilia length (**E**). Values are mean ± 2xSEM. *n* = 3 replicates of at least 300 cells. Statistical significance was determined using Student's t-test, *** = $p < 0.001$.

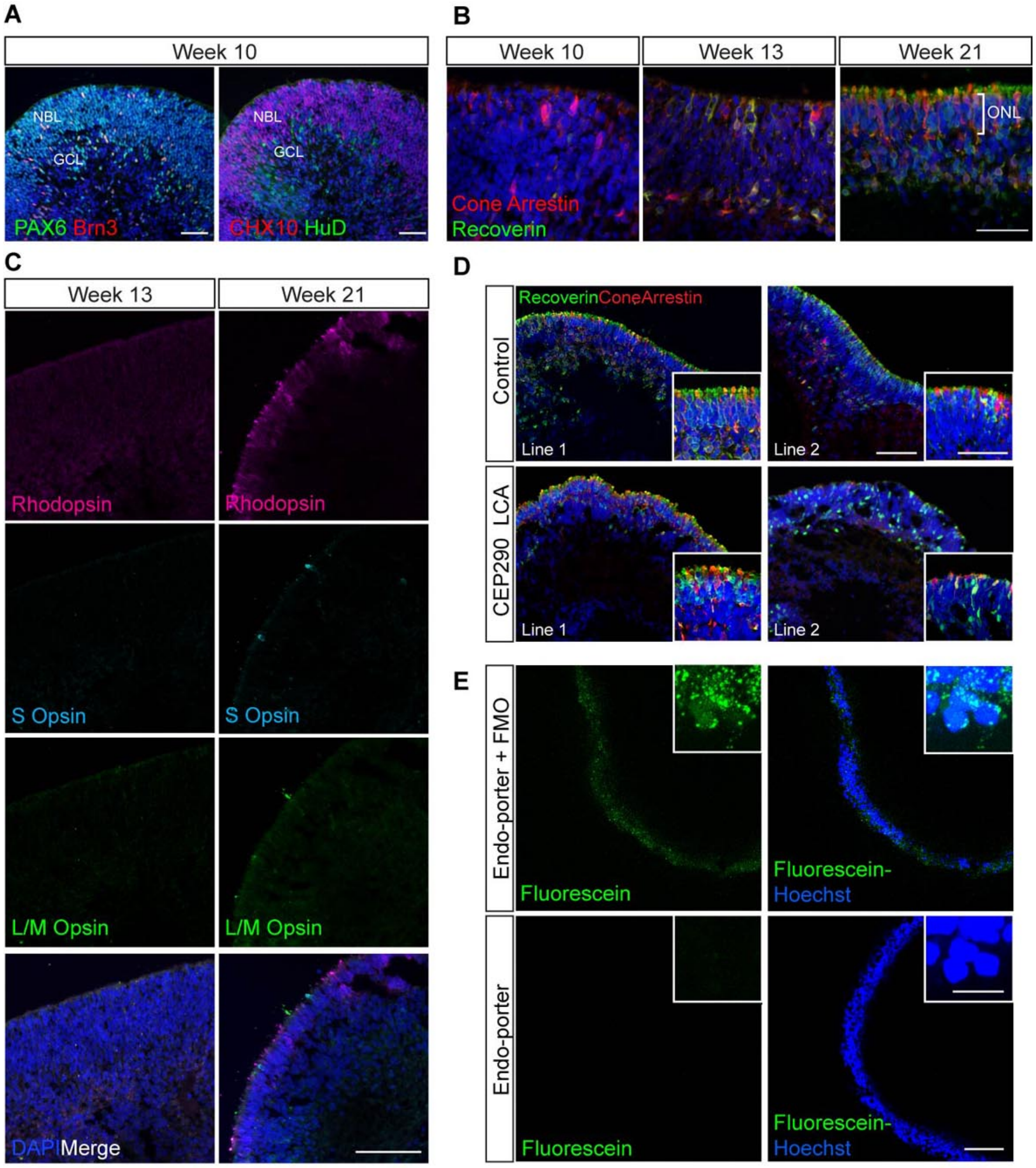


**Figure S4. Related to Figure 3 and 5. Optic cup development and MO accessibility.** (**A**) At week 10 optic cups had a thick transparent mantel and were bilaminated with a basal ganglion cell layer (GCL) expressing Brn3 and HuD, and a neuroblastic apical layer (NBL) expressing neural retinal progenitor markers Chx10 and Pax6. Scale bar = 50μm (**B**) Migrating recoverin (green) and cone arrestin (red) positive cells accumulated at the apical surface over time during optic cup maturation forming a defined outer nuclear layer (ONL). Scale bar = 50μm. (**C**) Low magnification triple stain for rhodopsin (magenta), S-opsin (cyan) and L/M-opsin (green) in control optic cups at weeks 13 and 21 showing the emergence and relative distribution of opsin expressing rods and cones. Scale bar = 100μm. (**D**) Low magnification images of optic cups derived from the four IPSC lines used in this study stained with recoverin (green) and cone arrestin (red). Control cell line 1 and 2 are from different individuals. LCA cell lines 1 and 2 are distinct clonal lines derived from the same CEP290 LCA patient harboring the c.2991+1665A>G mutation. Scale bar = 100μm; inset = 50μm (**E**) Live confocal imaging of Hoechst labeled optic cups 48 hours after treatment with Fluorescein labeled morpholino (FMO) or endoporter only control. Larger images show FMO penetrance through all cell layers. Inserts show dispersed fluorescence in the cytosol and nuclear compartment indicating successful delivery. Scale bars = 100μm; inset = 10μm.

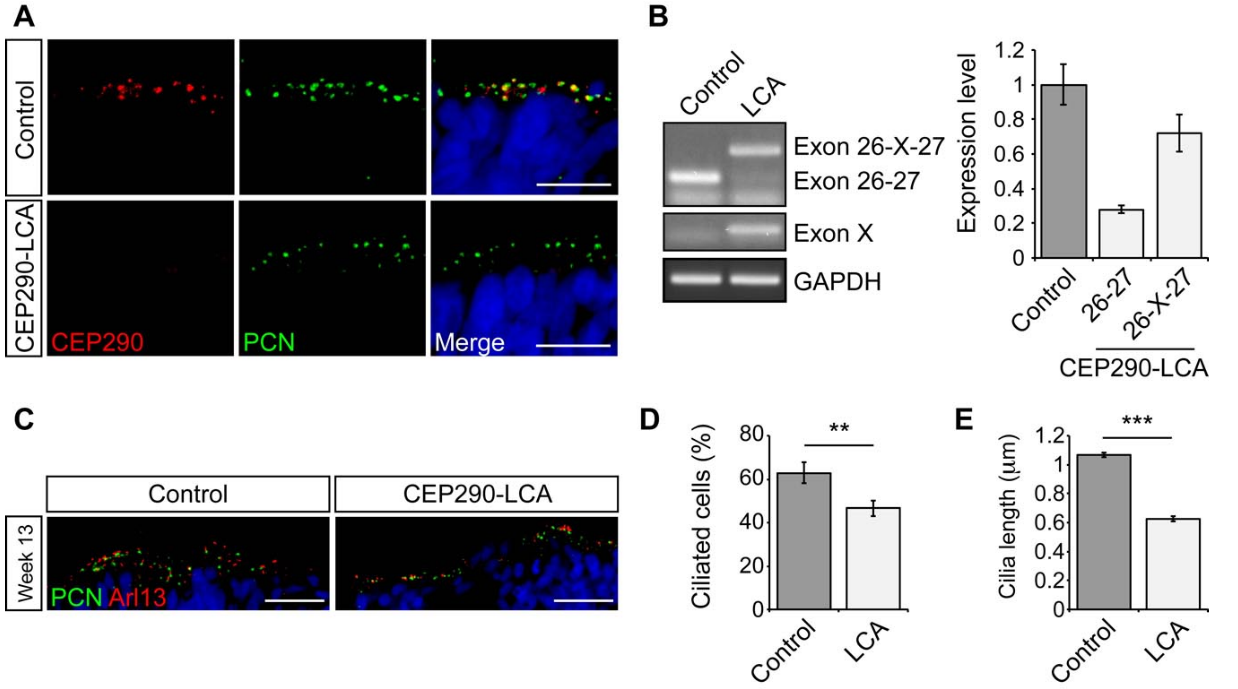


**Figure S5. Related to Figure 4. CEP290-LCA line 2 optic cups lack CEP290 expression and have decreased ciliation.** (**A**) CEP290 (red) expression was absent from the basal body (pericentrin (PCN), green) in LCA line 2 optic cups at week 13, compared to optic cups from control. Scale bar = 10µm. (**B**) RT-PCR analysis showed LCA optic cups had high relative levels of the cryptic exon. Values are mean ± 2xSEM. *n* = 3. (**C**) Representative images of immunostaining of cilia (Arl13, red; PCN, green) in control line 2 and CEP290-LCA line 2 optic cups at 13 weeks. Scale bar = 20µm. Quantification of ciliation (**D**) and cilia length (**E**) in 13 week CEP290-LCA line 2 optic cups, as determined by Arl13 and PCN staining compared to control line 2. Values are mean ± 2xSEM. *n* = 3 counts of 200 pericentrin-positive structures. Statistical significance was determined using Student's t-test, ** = $p < 0.01$, *** = $p < 0.001$.

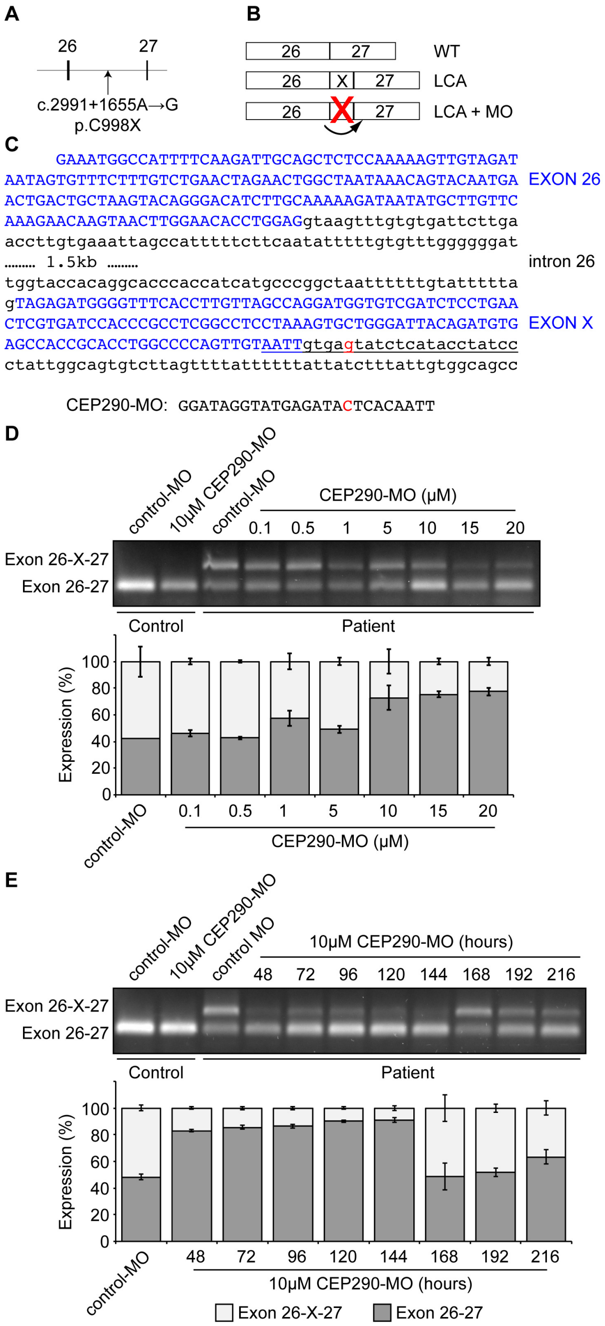


**Figure S6. Related to Figure 5. CEP290 morpholino validation.** (**A**) Gene structure of *CEP290* exons 26 and 27, showing location of the c.2991+1665A>G mutation deep in intron 27. (**B**) Schematic showing CEP290-MO method of action. In wildtype (WT) cells exon 26 and 27 are spliced normally. In CEP290-LCA cells, mis-splicing results in addition of cryptic exon (X). In LCA cells treated with CEP290-MO, the cryptic exon is skipped leading to restored normal transcripts. (**C**) Genetic sequence of the end of *CEP290* exon 26 (blue) and start of intron 26 (black) and addition of cryptic exon (exon X; blue). The mutation is marked in red and the sequence recognized by CEP290-MO is underlined. The sequence of CEP290-MO is shown underneath. (**D**) RT-PCR analysis of increasing doses of CEP290-MO in LCA fibroblasts. Control cells treated with CEP290-MO is also shown, revealing no negative effects of the MO on normal cells. Control-MO is used as a negative control in control and LCA cells. (**E**) RT-PCR analysis of increasing timepoints of 10µM CEP290-MO treatment on LCA fibroblasts. Values are mean ± 2xSEM.

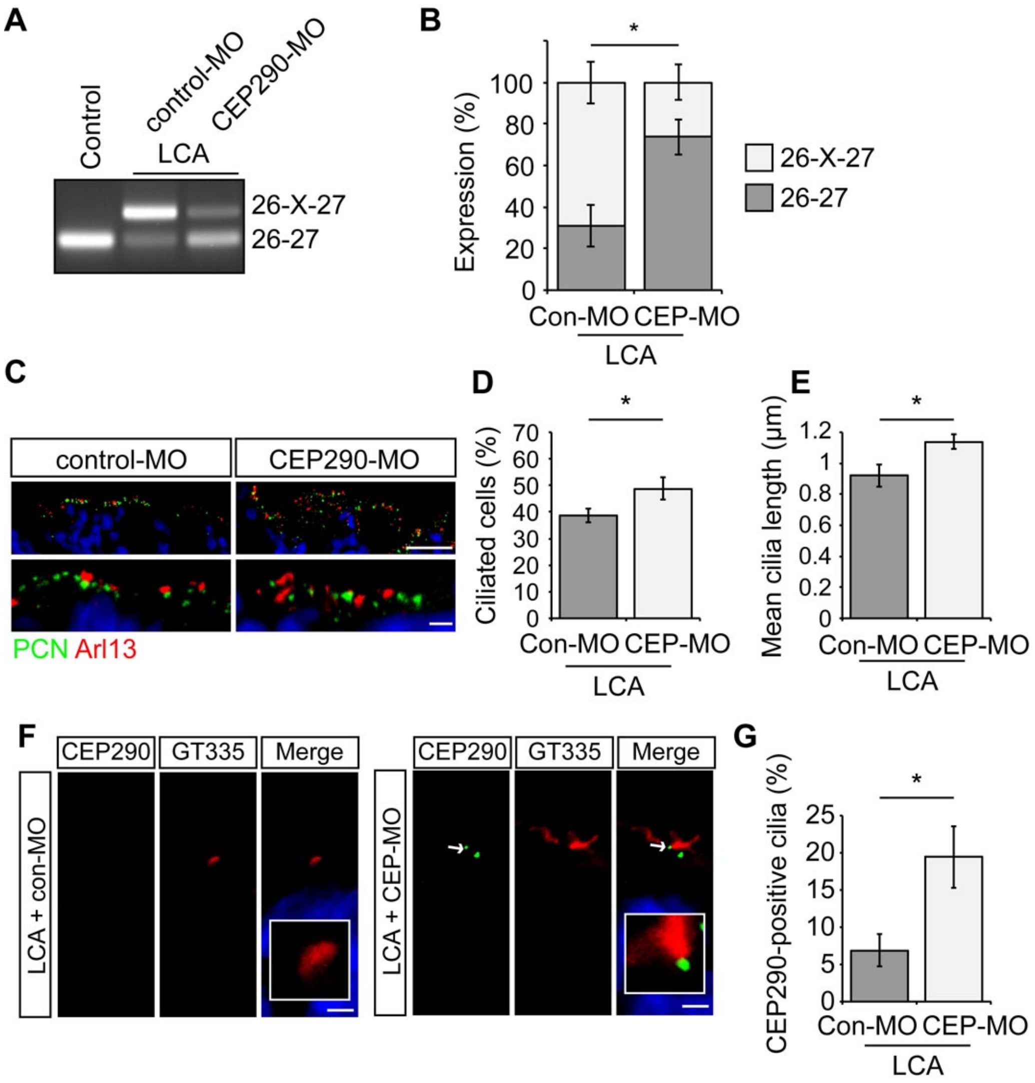


**Figure S7. Related to Figure 7. CEP290-MO treatment in LCA line 2 optic cups.** (**A**, **B**) RT-PCR analyses of *CEP290* exon 26-27 and quantification of bands in LCA line 2 optic cups. Values are mean ± 2xSEM. Statistical significance was determined using Student's t-test, * = $p < 0.05$, $n = 3$ for each treatment. (**C**) Representative images of cilia (Arl13, red; PCN, green) in LCA line 2 optic cups. Quantification of cilia incidence (**D**) and length (**E**) in LCA line 2 optic cups. Values are mean ± 2xSEM. Statistical significance was determined using Student's t-test, * = $p < 0.05$ $n = 5$ counts of at least 200 pericentrin-positive structures. Scale bar = 20µm (1µm in zoom panel). (**F**) CEP290 (green) localization at the connecting cilium (CC) in LCA optic cups. Ciliary axoneme is marked by polyglutamylated tubulin (GT335; red). Scale bar = 2µm. (**G**) Quantification of CEP290 localization at the CC. Values are mean ± 2xSEM. $n = 3$ replicates of at least 50 cilia. Statistical significance was determined using Student's t-test, * = $p < 0.05$.

**Supplemental Experimental Procedures**

**Reagents and antibodies**

Protease inhibitor cocktail (PIC), phosphatase inhibitor cocktail (PhIC) and 4',6-diamidino-2-phenylindole dihydrochloride (DAPI) were from Sigma. Anti-mouse cone-arrestin clone 7G6 (1:100) was the kind gift of Peter MacLeish, (Morehouse School of Medicine, Atlanta, GA, USA). Anti-rabbit CEP290 (1:100), anti-mouse pericentrin (1:1000), anti-Nanog (1:500) and anti-rabbit MerTK (1:500) were from Abcam. Anti-rabbit Arl13b (1:1000) was from ProteinTech. Anti-acetylated α-tubulin (1:1000), anti-rabbit Rab8 (1:100) and anti-rabbit RPGR were from Sigma, Dr Xinhua Shu (Glasgow Caledonian University) provided the anti-RPGR. Anti-mouse polyglutamylated tubulin GT335 (1:1000) was from Adipogen. Anti-mouse TRA-1-60 (1:1000) and anti-mouse CRALBP (1:500) were from Invitrogen. Anti-mouse rhodopsin 4D2 (1:500), anti-rabbit L/M opsin (1:5000) and anti-rabbit recoverin (1:500) were from Millipore. Anti-rabbit Pax6 (1:300) was from Covance. Anti-goat S-opsin (1:500), anti-goat Chx10, anti-rabbit HuD (1:300), anti-goat Brn3 (1:300) and anti-goat OTX2 (1:500) were from Santa Cruz. Anti-mouse Pmel17 (1:1000) was from Dako. Anti-mouse MitF (1:300) was from Neomarkers. Anti-rabbit ZO1 (1:300) was from Zymed. Anti-rabbit collagenase IV was from Biorad.

Goat anti-mouse and goat anti-rabbit Alexa Fluor 488 or Alexa Fluor 594 secondary antibodies conjugated IgGs (1:1000) were from Life Technologies. Donkey anti-goat Alexa Fluor 488 or Alexa Fluor 594 (1:500) were from Abcam. Horseradish peroxidase (HRP) conjugated donkey anti-rabbit or goat anti-mouse antibodies were from Pierce.

**Patient imaging**

Retinal fundus imaging was obtained by conventional 35 degree fundus color photographs (Topcon Great Britain Ltd, Berkshire, UK), 30 and 55 degree fundus autofluorescence (FAF) imaging, and spectral domain optical coherence tomography (OCT) scans (Spectralis, Heidelberg Engineering Ltd, Heidelberg, Germany).

**TaqMan hPSC Scorecard Panel**

Pluripotency potential was measured using TaqMan® hPSC Scorecard™ Assay (Thermo fisher, A15876). CEP290 iPSC cultures were split: half frozen in Trizol (TF 15596) after 4 days post passage and half used to make embryoid bodies after 6 days. The embryoid bodies were grown for a further 7 days before harvest (following Thermo Fisher publication MAN0008384). After Trizol RNA extraction, first strand synthesis was performed with the High Capacity cDNA RT Kit with RNaseInhibitor (Thermo Fisher) and qPCR performed in the using TaqMan® hPSC Scorecard™ Panel 96w FAST (Thermo Fisher) using TaqMan® Fast Advanced Master Mix (Thermo Fisher). Scorecard analysis was performed using Thermo Fisher's cloud-based software.

**Immunoblotting**

All samples were lysed on ice for 15 minutes using 1% *n*-dodecyl-β-D-maltoside (DM) buffer with 2% PIC and PhIC, separated by SDS-PAGE and analysed by western blotting. Primary antibodies were diluted in 5% BSA in Tris-buffered saline with 0.05% Tween20 (TBST) and incubation was carried out overnight at 4°C using the antibodies listed above, as indicated. Blots were developed using enhanced chemiluminescence (ECL) and scanned using the BioRad ChemiDoc system. Densitometry analysis was completed using ImageJ (http://rsbweb.nih.gov/ij/). The average pixel density was measured for each band.

**RNA extraction and RT-PCR**

Fibroblasts, RPE and optic cups were subjected to RNA extraction using RNeasy Mini Kit (Qiagen) and cDNA synthesis was performed using Tetro cDNA synthesis kit (Bioline) for reverse transcription. GoTaq Green (Promega) was used for amplification by PCR with standard cycling conditions. Primers used for PCR are listed below. Densitometry analysis was performed using ImageJ and the average pixel density was measured for each band. Levels of CEP290 bands were normalized to GAPDH and expressed relative to control levels.

**Immunofluorescence**

Cells were fixed in 4% paraformaldehyde for 10 minutes before permeabilization in 0.1% Triton X-100 for 10 minutes, at room temperature. RPE was fixed and cryosectioned as described previously (Schwarz et al., 2015). Optic cups were removed from culture media at specified time points and fixed in 4% paraformaldehyde at 4°C for 40 minutes. Post-fixation optic cups were cryoprotected by incubation overnight in 30% sucrose in PBS and then frozen and cyrosectioned. Fixed cells and cryosectioned RPE and optic cups were incubated in blocking buffer (3% bovine serum albumin (BSA) and 10% normal goat serum (NGS) in PBS) for 1 hour at room temperature before incubation with primary antibodies for 2 hours at room temperature, as indicated. Species-specific anti-IgG Alexa Fluor 488 or 594 secondary antibodies were used as appropriate. Nuclei were visualized using DAPI (2μg/ml) staining for all images. For cryosections, a final wash with 10mM copper sulphate in 50mM ammonium acetate (pH 5.0) was included to quench autofluorescence.

**Electron microscopy**

Optic cups were fixed overnight in a mixture of 3% glutaraldehyde and 1% paraformaldehyde buffered to pH 7.4

with 0.08M sodium cacodylate-HCl buffer. After rinsing in 0.1M sodium cacodylate-HCl buffer (pH 7.4) twice for 5 minutes, the optic cups were post-fixed in 1% aqueous osmium tetroxide for 2 hours, dehydrated by passage through ascending alcohols (10 minute steps, 1x 50-90% and 3 x 100%) and two changes of propylene oxide, and infiltrated overnight with 1:1 mixture of propylene oxide:araldite on a rotator. Finally optic cups were infiltrated with araldite resin over 4-6 hours and embedded in fresh resin, which was then cured by overnight incubation at 60°C. Semithin sections (0.75μm) were stained with a 1% mixture of toluidine blue-borax in 50% ethanol, and ultrathin sections were contrasted with Reynolds lead citrate for imaging in a JEOL 1010 TEM operating at 80kV. Images were captured using a Gatan Orius CCD camera using Digital Micrograph software.

**Assaying fluoresceinated morpholino delivery**

The delivery of morpholinos into optic cups was assayed using a morpholino oligo with a 3' carboxyfluorescein tag (GeneTools). Optic cups at week 13 were treated with 10μM fluoresceinated morpholino in 6μM endoporter (or endoporter only control). After 48 hours incubation, optic cups were washed, stained with Hoechst and imaged live on Zeiss LSM 510 inverted confocal microscope with heated stage.

**Imaging and statistical analysis**

All images were obtained using Carl Zeiss LSM700 laser-scanning confocal microscope. Images were exported from Zen 2009 software and prepared using Adobe Photoshop and Illustrator CS4. All measurements were performed in ImageJ. For cilia measurements, maximum intensity projections of z-stacks were used in the analysis. For fluorescence intensity at the basal body/cilia, double stained images were thresholded to select regions of interest containing basal body/cilia staining and the mean integrated fluorescence in the other channel was measured within these regions. A normal distribution was assumed for all data. For the statistical group analysis of fluorescence measurements and cilia counts in Figures 6 and 7, one-way analysis of variance (ANOVA) with *post-hoc* Tukey's test was used. For statistical analysis of all other measurements (RT-PCR quantifications and cilia measurements) Student's t-test was used. All statistical analysis was performed in either SPSS (version 22, IBM) or Microsoft Excel.

**Primer sequences**

| GENE | SEQUENCE (5'-3') |
|---|---|
| CEP290 26F | TGCTAAGTACAGGGACATCTTGC |
| CEP290 27R | AGACTCCACTTGTTCTTTTAAGGAG |
| CEP290 XF | CTGGCCCCAGTTGTAATTTGTGA |
| CEP290 XR | CTGTTCCCAGGCTTGTTCAATAGT |
| PAX6 F | TCAGCTCGGTGGTGTCTTTG |
| PAX6 R | GTCTCGGATTTCCCAAGCAA |
| VSX2/CHX10 F | AAGAAGCGGCGACACAGGACAATC |
| VSX2/CHX10 R | TTGGCTGACTTGAGGATGGACTCG |
| CRX F | CCCACTATTCTGTCAACGCCT |
| CRX R | TCTTGGCAAACAGTGCCTCC |
| NRL F | CACTGACCACATCCTCTCGG |
| NRL R | GAGGGTTCCCGCTTTACCTC |
| NR2E3 F | TGGTCCTCTTCAAGCCAGAGA |
| NR2E3 R | TTTCACCTCCACCCCCACTA |
| RPGR 14F | AGGGATTTTCATGACGCAGC |
| $RPGR^{ORF15}R$ | AGGTTCCATCCCCTCTACCT |
| RPGR 16R | CCTTTCTTCCTCCATGCACC |
| BBS8 1F | GGAGCTATTTTAGGCGCAGG |
| BBS2 2AR | ACTGGCAATTCAGGATCTGG |
| BBS8 3R | TTTCAAAGACGTTCCAGGGC |
| GAPDH F | TGCACCACCAACTGCTTAG |
| GAPDH R | GGATGCAGGGATGATGTTC |
